# Supplementary material for: Development of a new tool for the assessment of patient-defined benefit in hospitalised older patients: the Patient Benefit Assessment Scale for Hospitalised Older Patients (P-BAS HOP)
Source: BMJ Open. 2020 Nov 23;10(11):e038203. doi: 10.1136/bmjopen-2020-038203 (PMC7684819; doi:10.1136/bmjopen-2020-038203)
Supplement: Supplementary data [file bmjopen-2020-038203supp001.pdf]

Appendix 1. Version 1. Patient Benefit Assessment Scale

Hospitalisation goals

The following questions cover how important the goals below are for you during your current hospitalisation.

Can you indicate how important each goal below is for you? You can choose from: 'not at all', 'somewhat', 'moderately' 'quite', or 'very'. If a goal doesn't apply to you, for example because you don't have difficulty with the listed problem (for example with bowel movements, or shortness of breath) or because you don't have a garden, then choose 'does not apply to me'.

	How important is it for you that through this hospitalisation…	Not at all	Somewhat	Moderately	Quite	Very	Does not apply to me
1	You feel better						
2	You regain weight						
3	Your condition improves						
4	You have more energy						
5	You can walk better						
6	You move easier						
7	You can do housework						
8	You can cook						
9	You can do the groceries						
10	You can garden						
11	You can take pleasure in eating						
12	You can wash and dress yourself						
13	You can exercise or participate in sports						
14	You have no pain						
15	You have normal bowel movements						
16	You have less shortness of breath						
17	Your disease is under control						
18	You remain alive						
19	You can enjoy life						
20	You regain your freedom						
21	You can resume your (volunteer) work						
22	You can go on outings						
23	You can visit friends or family						
24	You can take a short break						
25	You know the cause of your complaints						
26	You can go back home						
27	You regain your independence						

Evaluation of hospitalisation goals

In the beginning of your hospitalisation you indicated how important various goals for you were. Can you indicate for each of the goals below how much the hospitalisation has helped to achieving the goal? You can indicate whether the hospitalisation helped you 'not at all', 'somewhat', 'moderately' 'quite', or 'very'. If a goal didn't apply to you, for example because you didn't have difficulty with the listed problem (for example with bowel movements, or shortness of breath) or because you have no garden, then choose 'did not apply to me'.

	The hospitalisation helped me to...	Not at all	Somewhat	Moderately	Quite	Very	Did not apply to me
1	Feel better						
2	Regain weight						
3	Improve my condition						
4	Have more energy						
5	Walk better						
6	Move easier						
7	Do the housework						
8	Cook						
9	Do the groceries						
10	Garden						
11	Take pleasure in eating						
12	Wash and dress myself						
13	Exercise or Participate in sports						
14	Have no pain						
15	Have normal bowel movements						
16	Have less shortness of breath						
17	Keep my disease under control						
18	Remain alive						
19	Enjoy life						
20	Regain my freedom						
21	Resume my volunteer work						
22	Go on outings						
23	Visit family or friends						
24	Can take a short break						
25	Know what the cause of my complaints is/was						
26	Go back to my home						
27	Regain my independence						
