# Supplementary material for: Development of a new tool for the assessment of patient-defined benefit in hospitalised older patients: the Patient Benefit Assessment Scale for Hospitalised Older Patients (P-BAS HOP)
Source: BMJ Open. 2020 Nov 23;10(11):e038203. doi: 10.1136/bmjopen-2020-038203 (PMC7684819; doi:10.1136/bmjopen-2020-038203)
Supplement: Supplementary data [file bmjopen-2020-038203supp002.pdf]

Appendix 2. Final Version Patient Benefit Assessment Scale

Hospitalisation Goals

People differ in what they wish to achieve with a hospitalisation. They have different goals. This depends on what they suffer from, what they are hospitalised for, and what they find important in life.
I am now going to mention some subjects that may be important to you during this hospitalisation. Can you say whether each applies to you?
A subject applies to you if you experience or anticipate problems or limitations and this applies to your life. For people who, for example, are short of breath, the subject shortness of breath probably applies, but not for others. For others they may be struggling to enjoy life because of their illness, but if you experience no problems with it, then this doesn't apply to you.

Instruction for the interviewer: Circle the number for the subject that applies to the participant.

If a participant asks what you mean by a subject, say:
Are you experiencing problems with [subject] now, or when you were admitted, or are you expecting problems with it because of your illness or hospitalisation?
For the functional subjects, prefer not to use the word 'problem', but rather 'trouble' or 'limitation'.

Now follow the subjects, you should indicate whether each subject applies to you or not:
1. Wanting to feel better
Explanation if necessary: do you feel sick or miserable and would you like to feel better?
2. Energy
3. Pain
4. Bowel movements
5. Shortness of breath
6. Walking
7. Appetite
8. Unclarity about what is wrong with me
Explanation if necessary: you may be wondering what is wrong with you. If this was already clear for you when you were admitted then this does not apply.
9. Controlling my disease
Explanation if necessary: Do you suffer from a disease that needs to be controlled?
10. Remain alive
Explanation if necessary: Was your life in danger when you were admitted or do you think that this hospitalisation must contribute to remain alive?
11. Enjoying life
12. Groceries
13. Washing and dressing myself
14. Gardening
15. Exercise or Sports
16. Hobbies
17. Driving
18. Going on outings
19. Visiting family or friends

20. Return back to my home
Explanation if necessary: Are you unsure whether you can return to your own home?
21. Independence
22. Are there other themes which are important to you during this hospitalisation that I have not mentioned yet? If so, which?

Now that we have identified the themes that apply to you, I am going to ask to what extent they are goals during this hospitalisation.

Can you indicate for each goal that I mention how important it is during this hospitalisation? You can choose from 'not at all important', 'moderately important' 'quite important', or 'very important'.

For some goals you may still say that they do not apply, for example because they do not matter during this hospitalisation. Then you indicate "does not apply to me now".

Instruction to interviewer: Read only the goals which you have circled.

		does **not apply** to me now	**not at all** important	**somewhat** important	**quite** important	**very** important
1	How important is it for you that you **feel better again** as a result of this hospitalisation	☐	☐	☐	☐	☐
2	How important is it for you that you **have more energy** as a result of this hospitalisation	☐	☐	☐	☐	☐
3	How important is it for you that you **have less pain** as a result of this hospitalisation	☐	☐	☐	☐	☐
4	How important is it for you that you **have normal bowel movements again** as a result of this hospitalisation	☐	☐	☐	☐	☐
5	How important is it for you that you **are less short of breath** as a result of this hospitalisation	☐	☐	☐	☐	☐
6	How important is it for you that you can **walk better again** as a result of this hospitalisation	☐	☐	☐	☐	☐
7	How important is it for you that you **regain your appetite** as a result of this hospitalisation	☐	☐	☐	☐	☐
8	How important is it for you that you **know what is wrong with you** as a result of this hospitalisation	☐	☐	☐	☐	☐
9	How important is it for you that **your disease is under control** as a result of this hospitalisation	☐	☐	☐	☐	☐
10	How important is it for you that you **remain alive** as a result of this hospitalisation	☐	☐	☐	☐	☐
11	How important is it for you that you can **enjoy life again** as a result of this hospitalisation	☐	☐	☐	☐	☐

		does **not apply** to me now	**not at all** important	**somewhat** important	**quite** important	**very** important
12	How important is it for you that you can **do the groceries again** as a result of this hospitalisation	☐	☐	☐	☐	☐
13	How important is it for you that you **can wash and dress yourself again** as a result of this hospitalisation	☐	☐	☐	☐	☐
14	How important is it for you that you **can garden again** as a result of this hospitalisation	☐	☐	☐	☐	☐
15	How important is it for you that you **can exercise or participate in sports again** as a result of this hospitalisation	☐	☐	☐	☐	☐
16	How important is it for you that you **can exercise your hobbies again** as a result of this hospitalisation	☐	☐	☐	☐	☐
17	How important is it for you that you **can drive again** as a result of this hospitalisation	☐	☐	☐	☐	☐
18	How important is it for you that you **go on outings again** as a result of this hospitalisation	☐	☐	☐	☐	☐
19	How important is it for you that you **can visit family or friends again** as a result of this hospitalisation	☐	☐	☐	☐	☐
20	How important is it for you that you **can return to your own home again** as a result of this hospitalisation	☐	☐	☐	☐	☐
21	How important is it for you that you **regain your independence** as a result of this hospitalisation	☐	☐	☐	☐	☐
22	How important is it for you that you ____________________ as a result of this hospitalisation	☐	☐	☐	☐	☐
23	Do you have any goals I haven't mentioned yet? If so: How important is it to you that____________________________ as a result of this hospitalisation	☐	☐	☐	☐	☐

Evaluation of hospitalisation goals

In the beginning of your hospitalisation you indicated how important various goals for you were. A goal is something you want to achieve with a hospitalisation. Some goals you may have achieved, others maybe not or not entirely.

Can you indicate for each of the goals below how much the hospitalisation has helped to achieving the goal? You can indicate whether the hospitalisation has helped you 'not at all', 'moderately', 'quite', or 'completely'.

Only the goals which applied at baseline are evaluated with the participant.

		Not at all	Somewhat	Quite	Completely
1	Because of the hospitalisation I **feel better again**	☐	☐	☐	☐
2	Because of the hospitalisation I have **more energy**	☐	☐	☐	☐
3	Because of the hospitalisation I have **no more pain**	☐	☐	☐	☐
4	Because of the hospitalisation I have **normal bowel movements again**	☐	☐	☐	☐
5	Because of the hospitalisation I am **less short of breath**	☐	☐	☐	☐
6	Because of the hospitalisation I **walk better again**	☐	☐	☐	☐
7	Because of the hospitalisation I **regained appetite**	☐	☐	☐	☐
8	Because of the hospitalisation I **know what is/ was wrong with me**	☐	☐	☐	☐
9	Because of the hospitalisation **my disease is under control**	☐	☐	☐	☐
10	Because of the hospitalisation I **remained alive**	☐	☐	☐	☐
11	Because of the hospitalisation I **enjoy life again**	☐	☐	☐	☐
12	Because of the hospitalisation I **do the groceries again**	☐	☐	☐	☐
13	Because of the hospitalisation I **wash and dress myself again**	☐	☐	☐	☐
14	Because of the hospitalisation I **garden again**	☐	☐	☐	☐
15	Because of the hospitalisation I **participate in sports again**	☐	☐	☐	☐

		Not at all	Somewhat	Quite	Completely
16	Because of the hospitalisation I **can resumed my hobbies**	☐	☐	☐	☐
17	Because of the hospitalisation I **drive again**	☐	☐	☐	☐
18	Because of the hospitalisation I **go on outings again**	☐	☐	☐	☐
19	Because of the hospitalisation I **visit family or friends again**	☐	☐	☐	☐
20	Because of the hospitalisation I **am back in my own home**	☐	☐	☐	☐
21	Because of the hospitalisation I **regained independence**	☐	☐	☐	☐
22	Because of the hospitalisation ______________________	☐	☐	☐	☐
