# Supplementary material for: Development of a new tool for the assessment of patient-defined benefit in hospitalised older patients: the Patient Benefit Assessment Scale for Hospitalised Older Patients (P-BAS HOP)
Source: BMJ Open. 2020 Nov 23;10(11):e038203. doi: 10.1136/bmjopen-2020-038203 (PMC7684819; doi:10.1136/bmjopen-2020-038203)
Supplement: Author's manuscript [file bmjopen-2020-038203.draft_revisions.pdf]

BMJ Open is committed to open peer review. As part of this commitment we make the peer review history of every article we publish publicly available.

When an article is published we post the peer reviewers' comments and the authors' responses online. We also post the versions of the paper that were used during peer review. These are the versions that the peer review comments apply to.

The versions of the paper that follow are the versions that were submitted during the peer review process. They are not the versions of record or the final published versions. They should not be cited or distributed as the published version of this manuscript.

BMJ Open is an open access journal and the full, final, typeset and author-corrected version of record of the manuscript is available on our site with no access controls, subscription charges or pay-per-view fees (http://bmjopen.bmj.com).

If you have any questions on BMJ Open's open peer review process please email info.bmjopen@bmj.com

BMJ Open

Development of a new tool for the assessment of patient defined benefit in hospitalised older patients: the Patient Benefit Assessment Scale for Hospitalised Older Patients (P-BAS HOP)

Journal:	*BMJ Open*
Manuscript ID	bmjopen-2020-038203
Article Type:	Original research
Date Submitted by the Author:	03-Mar-2020
Complete List of Authors:	van der Kluit, Maria; University of Groningen, University Medical Center Groningen, University Center for Geriatric Medicine<br>Dijkstra, Geke; Department of Health Sciences, Applied Health Research<br>van Munster, Barbara; University of Groningen, University Medical Center Groningen, University Center for Geriatric Medicine; Gelre Hospitals, Geriatrics<br>De Rooij, Sophia; University Medical Center Groningen, University Center for Geriatric Medicine; Medical Spectrum Twente, Medical School Twente
Keywords:	GERIATRIC MEDICINE, QUALITATIVE RESEARCH, Quality in health care < HEALTH SERVICES ADMINISTRATION & MANAGEMENT

SCHOLARONE™
Manuscripts

I, the Submitting Author has the right to grant and does grant on behalf of all authors of the Work (as defined in the below author licence), an exclusive licence and/or a non-exclusive licence for contributions from authors who are: i) UK Crown employees; ii) where BMJ has agreed a CC-BY licence shall apply, and/or iii) in accordance with the terms applicable for US Federal Government officers or employees acting as part of their official duties; on a worldwide, perpetual, irrevocable, royalty-free basis to BMJ Publishing Group Ltd ("BMJ") its licensees and where the relevant Journal is co-owned by BMJ to the co-owners of the Journal, to publish the Work in this journal and any other BMJ products and to exploit all rights, as set out in our licence.

The Submitting Author accepts and understands that any supply made under these terms is made by BMJ to the Submitting Author unless you are acting as an employee on behalf of your employer or a postgraduate student of an affiliated institution which is paying any applicable article publishing charge ("APC") for Open Access articles. Where the Submitting Author wishes to make the Work available on an Open Access basis (and intends to pay the relevant APC), the terms of reuse of such Open Access shall be governed by a Creative Commons licence – details of these licences and which Creative Commons licence will apply to this Work are set out in our licence referred to above.

Other than as permitted in any relevant BMJ Author's Self Archiving Policies, I confirm this Work has not been accepted for publication elsewhere, is not being considered for publication elsewhere and does not duplicate material already published. I confirm all authors consent to publication of this Work and authorise the granting of this licence.

For peer review only

Development of a new tool for the assessment of patient defined benefit in hospitalised older patients: the Patient Benefit Assessment Scale for Hospitalised Older Patients (P-BAS HOP)

Authors:

1. Maria Johanna van der Kluit, MSc RN (Corresponding author)

   University of Groningen, University Medical Center Groningen, University Center for Geriatric Medicine, Hanzeplein 1, 9700 RB Groningen, The Netherlands

   m.j.van.der.kluit@umcg.nl

   +31503613921

2. Geke J. Dijkstra, PhD

   University of Groningen, University Medical Center Groningen, Department of Health Sciences, Applied Health Research, Groningen, The Netherlands

   g.j.dijkstra@umcg.nl

3. Barbara C. van Munster, MD PhD

   University of Groningen, University Medical Center Groningen, University Center for Geriatric Medicine, Groningen, The Netherlands.

   Department of Geriatrics, Gelre Hospitals, Apeldoorn, The Netherlands.

   b.c.van.munster@umcg.nl

4. Sophia E. de Rooij, MD PhD

   University of Groningen, University Medical Center Groningen, University Center for Geriatric Medicine, Groningen, The Netherlands

   Medical Spectrum Twente, Medical School Twente, Enschede, The Netherlands

   sejaderooij@gmail.com

Words: 5003

Words abstract: 298

References: 22

Figures: 1

Tables: 4

For peer review only

ABSTRACT

Objectives: To support the shift from disease-oriented towards goal-oriented care, we aimed to develop a tool which is capable both to identify priorities of an individual older hospitalised patient and to measure his relevant outcomes.

Design: Mixed methods design with open interviews, Three Step Test Interviews (TSTI), and a quantitative field test.

Setting: University teaching hospital and a regional teaching hospital.

Participants: Hospitalised patients ages 70 years and older.

Results: The Patient Benefit Assessment Scale for Hospitalised Older Patients (P-BAS HOP) consists of a baseline questionnaire and an evaluation questionnaire. Items were based on qualitative interviews with hospitalised older patients. Feedback from a panel of four community-dwelling older persons resulted in some adaptations to wording and one additional item. TSTIs with Version 1 of the baseline questionnaire with twenty-six hospitalised older patients showed indications for a good content validity and revealed barriers in completion behaviour, global understanding, and understanding of individual items, which were solved with several adaptations. Four additions were made by participants. After TSTIs with ten patients with the evaluation questionnaire, one adaptation was made. A field test with 91 hospitalised older patients revealed a small number of missing values.

To enhance the feasibility, the number of items was reduced based on correlations and mean impact score. The field test was repeated with 104 patients in a regional teaching hospital. To enhance the understanding, the tool was split into two phases. This version was tested with TSTIs with eight patients and appeared to be understandable. The final version was an interview-based tool with 22 items plus an open option and took about 11 minutes to complete.

Conclusions: The P-BAS HOP is a potentially suitable tool to identify priorities and relevant outcomes of the individual patient. Further research is needed to investigate its validity, reliability and responsiveness.

Keywords Geriatric medicine; Older adults; Hospitalisation; Patient perspective; Goal setting; Patient-reported outcomes; Tool; Value-Based Health Care

ARTICLE SUMMARY

Strengths and limitations of this study

- The content of the P-BAS HOP is based on open interviews with hospitalised older patients.
- Patients are able to indicate their individual outcome priorities.

For peer review only

- The P-BAS HOP is tested intensively in the target population with Three Step Test Interviews. This gave valuable insights into the understanding of the tool and the completion behaviour of the participants.
- The current version of the P-BAS HOP is only suitable to be completed with an interviewer and not as a self-administered questionnaire.
- It is unknown whether the P-BAS HOP is feasible in other healthcare systems, languages and cultures than in the Netherlands.

For peer review only

BACKGROUND

To fit the needs of the ageing population, and patients with multiple chronic diseases, a shift is recommended from disease-oriented towards goal-oriented care. Older patients with multimorbidity may be more interested in more personal goals such as for them important symptoms, functional status and social functioning than in traditional outcomes such as survival and biomarkers.[1, 2] When care would be systematically evaluated by personal goal-oriented outcomes, a tool is needed which is capable both to identify the priorities of the individual patient and to measure his relevant outcomes.

Three literature reviews[3-5] into tools used to assess patient outcome priorities in the context of multimorbidity revealed a few potentially useful tools. Tools only suitable for specific activities, such as the Canadian Occupational Performance Measure (COPM),[3, 5] Self-Identified Goals Assessment (SIGA),[5] Assessment of Motor and Process Skills (AMPS) and McMaster Toronto Arthritis (MACTAR)[3] were ignored. A general tool is the Outcome Prioritization Instrument,[4, 6] which is suitable to elicit four patient priorities, but these priorities are still very global and it remains unclear how to evaluate them after treatment. Another tool is the Goal Attainment Scaling (GAS)[3, 5] which is designed to set and evaluate individualized goals and outcomes. Disadvantages of the GAS are that it can be too challenging for patients to articulate their own goals and that it is time consuming.[7] The International Classification of Functioning, Disability and Health (ICF) framework for goal setting is used to categorize patient goals set in semi-structured interviews, but has still the same disadvantages as the GAS and has, in addition, a very poor responsiveness.[5] Finally, with the Target Complaints,[3] the patient defines target complaints as those problems for which help was sought. These complaints are scored at the beginning and at the end of the treatment by the patient, or the patient rates the degree of improvement.[8, 9] The Target Complaints is individualized and patient-centred. However, it focuses solely on problems and not on goals. Disadvantages for the GAS, ICF and Target Complaints could be that for some older patients it might be difficult to formulate their own goals and problems because many older persons are not accustomed to defining and discussing personal goals and prompting is often necessary.[10] The quality of the answers is therefore dependent on the interviewer's experiences and techniques.

For this reason, another method of defining patient-defined goals and outcomes was sought and found in the literature about treatment of acne. Augustin et al.[11] developed a tool consisting of two parts: 1) a baseline questionnaire to assess the importance of various predefined goals, based on themes derived from qualitative interviews in patients with acne and 2) an evaluation questionnaire to evaluate the extent to which treatment helped to achieve these goals. Based on these data it is possible to compute an individual Patient Benefit Index. This is an overall value between 0 (no benefit) and 4 (maximal benefit), which reflects the achievement of the goals weighted by the

For peer review only

importance.[11] The advantage of this tool is the insight into the individualized patient perspective, together with standardisation.

The aim of this study was to develop a tool to inventory individual goals and benefits of older hospitalised patients, based on the model of Augustin et al.[11] This article presents its development, early testing and adaptations.

METHODS

The steps used to develop the Patient Benefit Assessment Scale for Hospitalised Older Patients (P-BAS HOP) are based on the steps of De Vet et al.[12] and outlined in Figure 1. After each step, the tool was adapted. The steps are explained in the following sections. The P-BAS was developed and tested in Dutch. The P-BAS was translated into English in a translation - back translation procedure involving four translators (two native English, two native Dutch), a language professional and authors MJvdK and GD.[13]

Figure 1. Development of the P-BAS HOP

1. Qualitative study

Firstly, open interviews with hospitalised older medical and surgical patients about their goals regarding their hospitalisation were performed. The description of these goals is published elsewhere.[14] These goals were then coded inductively and transformed into questionnaire items, and the first draft of the P-BAS HOP was then constructed, consisting of a baseline questionnaire and an evaluation questionnaire.

2. Panel of community-dwelling older persons

The first draft version was proposed by e-mail to a panel of community-dwelling older persons to assess the comprehensibility and relevance of the items and the tool and ask for omissions or redundancies.

3. Pilot test: Three Step Test Interview (TSTI).

The adapted tool (Version 1, Appendix 1) was tested with the TSTI[15, 16] in older hospitalised patients. The TSTI is a type of cognitive interview suitable to assess how people interpret a questionnaire, its different items and what kind of strategies they use in responding to them. The TSTI consists of the following steps:

Step 1: Concurrent thinking aloud

The participant completed version 1 of the P-BAS HOP while thinking aloud. The interviewer observed, made notes of the participant's behaviour (hesitations, skipping questions, corrections) and verbalized thoughts. However, the interviewer did not talk, or intervene. The instructions for the

For peer review only

participant were: Please fill in this questionnaire and try to think aloud about what your thoughts are while reading the questions and choosing the right response category.

Step 2: Retrospective interview

With the retrospective interview any gaps from the first step were filled in. Every behaviour and thought from the observation of which the interviewer wanted further information, was clarified.

Step 3: Semi-structured interview

An in-depth interview was conducted, aimed at eliciting the participant's considerations and opinions. The participant was given the opportunity to explain behaviour, actions or thoughts that he had in the previous steps. The participant was asked how he understood different items, was asked for any omissions and his opinion about filling in the questionnaire. The participant was also asked to explain his goals in his own words in order to perform a first content validation of the P-BAS HOP.

Participants

Eligible participants of the TSTI were 70 years and older; planned or unplanned hospitalised on medical or surgical wards of a university teaching hospital in the Netherlands; able to speak and understand Dutch and were without cognitive impairment. Inclusion criteria were verified with the staff nurse, and patients were then approached by the interviewer (MJvdK). Participants were completely anonymous, no list with names or other identifying data was made, nor did the researchers have access to medical records. Participants gave verbal consent to the interview and audio recording.

Data analysis

Data gathering and data analysis were alternated. Interviews were audio recorded and transcribed verbatim. All remarks were then organised by question and step. After that, the data were coded by MJvdK and grouped into categories. The tool was adapted several times after the feedback until it was considered feasible and understandable.

The TSTI was repeated with the evaluation questionnaire. This was done at patient discharge. These steps led to construction of the P-BAS HOP Version 2.

4. Field test with Version 2. Item reduction based on mean impact score and correlation.

Version 2 was tested with a new group of hospitalised older patients. The aim of this field test was to assess the feasibility of the P-BAS HOP in combination with other questionnaires. The trained research assistants observed during the field test that the tool was too time consuming and that some patients still had difficulties relating the questions to their personal situation, as was observed

For peer review only

in the TSTI. Therefore, the following extra adaptations were made: item reduction, answer option reduction, and splitting the tool into two phases.

Participants

Eligible participants were consecutive patients aged 70 years and older; planned or unplanned hospitalised on medical or surgical wards of a university teaching hospital, expected to stay for at least 48 hours; and at maximal four days hospitalised at the moment of interviewing; able to speak and understand Dutch and were without cognitive impairment. Inclusion criteria were verified with the staff nurse. Patients were approached by a trained research assistant and gave signed informed consent to participate. The questionnaire was then conducted in a face to face interview with the research assistant, but to patients in a better condition and with middle or higher education the opportunity was given to fill in the questionnaire themselves, an option which only a minority of patients choose.

Item reduction

Item reduction was based on correlation and mean impact score.

Items within one category with a strong correlation, measured probably the same construct. Therefore, from dyads with a Spearman's rank-order correlation > 0.7, one item was removed.[12] For the calculation of the Spearman's rank-order correlation coefficient, the answer option 'does not apply to me now' and 'not at all important' were coded as 0, the options somewhat, moderately, quite important and very important were coded respectively as 1, 2, 3, 4.

For the reduction based on mean impact score, all items were sorted into categories. For each item the mean impact score was calculated: [% for whom the item played a role] * [mean importance for that item]. From every category with two or more items, the item with the lowest mean impact score was removed.[12] The field test was repeated in a regional teaching hospital by a trained research assistant, to check whether the impact differed in another context.

5. TSTI with Version 3.

Version 3 was tested again with the TSTI in hospitalised older patients. The procedure was identical as in step 3, however as this version is only applicable as an interview version, this was done with an interviewer and observant. The observant only observed during the first step, and took over the interview role in the second and third steps.

Patient and public involvement

For peer review only

Patients and public were involved in the generation of the items, the importance and relevance of the items and the assessment of the feasibility and understanding of the tool.

RESULTS

1. Qualitative study

After the first fifteen qualitative interviews, the goals formulated in these interviews were transferred to questionnaire items, which resulted in the first draft. The results of this qualitative study are published elsewhere.[14]

2. Panel of community-dwelling older persons

Four community-dwelling older persons gave written feedback on the draft version of the tool. This led to adaptation in wording of the introduction text and to some items which were not clear enough or could be interpreted in multiple ways. An example of an item that was adapted: for the item 'to be able to eat', it was not clear if it concerned the instrumental activity of eating or concerned appetite. Therefore, the item was changed into 'can take pleasure in eating'.

Further, one additional item was added, namely 'to wash and dress yourself' and the sequence of two items was changed. Version 1 of the P-BAS HOP is shown in Appendix 1.

3. TSTI

Sample baseline questionnaire

Twenty-six older hospitalised patients participated in the TSTI. Characteristics of the participants are displayed in the second column of Table 1.

Table 1. Participants Three Steps Test Interview (TSTI) baseline questionnaire version 1, evaluation questionnaire version 1 and baseline questionnaire version 3.

	Baseline questionnaire version 1 (n=26)	Evaluation questionnaire version 1 (n=10)	Baseline questionnaire version 3 (n=8)
Characteristic	n	n	n
Gender			
Male	19	8	4
Female	7	2	4
Age (years)			

For peer review only

70-79	18	8	7
80-89	7	2	0
90-99	1	0	1
Native language			
Dutch	14	7	7
Local dialect	10	2	1
Frisian	2	0	0
Foreign language	0	1	0
Educational level*			
Low	8	5	2
Middle	10	4	5
High	8	1	1
Admission reason†			
Cardiac problems	9	1	1
Pulmonary problems	7	3	2
Bowel problems	2	2	1
Fever/ infection	2	2	0
Vascular surgery	2	1	0
Cancer	2	0	0
Accident/fracture(s)	2	0	1
Kidney problems	0	1	1
Syncope	0	0	1
Ulcera	0	0	1

* Definition educational level: Low= no education, primary school, basic vocational training; Middle = secondary education, vocational training; High = bachelor, master

† Reason according to the patient

Coding categories

The codes were sorted into the following categories: Completion behaviour, Global understanding, Understanding and reactions regarding individual items, Additions, General evaluation, and Content validity.

Completion behaviour

For peer review only

With ‘completion behaviour’ is meant the behaviour participants showed when completing the questionnaire. Observations revealed that many participants skipped the instruction text partly or even completely. For some participants, reading these instructions was demanding, others did not understand how a table works and where to place a mark. Adding an example table with instructions showing how and where to place a mark brought no improvements, since some participants started to complete the example, although ‘example’ was indicated very explicitly in the table. In the final version boxes to tick were included in the table.

Global understanding

Many examples of correct understanding were coded. For example:

> Item *regaining weight*, step 1: *Oops, dear, pooh, let's see, that is certainly important, because I have lost weight lately since I had not been feeling well for a while, that is, not shortness of breath or anything, but, very tired, listless. So, a little weight gain is important. I came from [other hospital], where I was first admitted and so I got help here, and there I already got a little bottle of nutritional drink twice a day, which I, yes, I always call it baby food, to strengthen myself. Lots of calories, proteins, etc. So that is important. (P9)*
>
> Or:
>
> Item *walking*, step 1: *Well, I walk well. Doesn’t apply to me.* (*P17*)

A few participants interpreted the questions as if it was an evaluation of their current level of functioning. For example:

> Item *energy*, step 2: *I: You have filled in ‘not at all’ in ‘you have more energy’. What is the reason that you just ...? P: Because I feel lethargic. That is what I mean to say. I used to be a very energetic person. I was talking about it with my son and my wife last week: I could do everything, I did everything and I tried everything. That is gone. I: That's gone, yes. P: That is what I mean by that question. I: Yes, so you actually filled in how you are feeling now. P: Yes, now. At the time. I: Yes, so you say .. P: Not from last year or half a year ago. They are snapshots, aren't they? That was what you meant, right? (...) I: And when I ask you the question: ‘How important is it that you get energy again?’ P: Very important. I: That's very important. P: Because I've always been energetic. Very important. I: Yes, okay. P: Terribly important.* (*P2*)

Other participants had difficulties relating the goals to their own situation. For example:

> Item *shortness of breath*, step *1 I'm actually never short of breath. But it is quite important.* (*P25*)

For peer review only

There were also participants who did recognize that a certain goal did not apply for them, but they did not understand how to indicate that in the tool. For example:

> Item *regaining weight*, step 2: *I: I see that you have left open question 2, about regaining weight. P: Oh, that, uh, regain weight. (…) I: So I wondered does that have a special reason that you left it open? P: I don't have to regain weight. I don't have to. I have to go down. See, that is not there. So, what am I supposed to do with that, I don't, I don't know what… I: Then you don't know what to do with it. P: No. Then I think, I just continue.* (*P18*)

Some participants were reluctant to use the options 'not at all important' or 'doesn't apply to me', because they deemed those answers socially undesirable.

Since many older persons have multiple health problems, it is possible that a participant experiences a problem with an item, but is admitted for another health problem. Many participants were able to make this distinction. For example:

> Item *moving*, step 1: *Yes, that will never be all right again, I can tell. Does no longer apply at all. Already 30 years ago they said: Mr. B., you have to learn to live with that. And they still say that today. Osteoarthritis, there is nothing you can do about it.* (*P18*)

But for others this distinction was more difficult.

Adaptations

To enhance the general understanding, the following adaptations to the tool were made and tested in new participants:

Several adaptations were made in the instruction text.

In the columns with the answer options the word 'important' was added to all answer options. For example: 'very' was changed into 'very important', to make clear that the question was not to evaluate current function, but to indicate how important the goal was.

The sequence of the questions was changed. To enable participants to relate the goals to their own situation, the questions related to somatic complaints were moved to the beginning of the questionnaire.

Another adaptation made to improve the understanding to relate the goals to the patients' own situation, was to add the word 'again' to the goals, to make clear that it is something they had before and they have to regain by the hospital admission. For example: *How important is it to you that you have normal bowel movements again*.

The next adaptation was to move the answer option 'doesn't apply to me' from the last to the first column. This made it easier to find that option.

For peer review only

The final adaptation to improve making the connection between the hospital admission and their goals, was repeating the question in every line. Instead of having the text '*How important is it to you that by this hospital admission…'* on top of the page alone, this question was repeated in every row. Apart from this, several adaptations were made to the layout in order to ease the reading for participants.

Understanding and reactions regarding individual items

The following individual items caused discussion: take pleasure in eating, to know the cause of your complaints, take a short break, and remain alive.

Take pleasure in eating

Some participants had a more epicurean association with this item. Therefore, it was changed into: 'regain appetite'.

Cause of complaints

With the item 'how important is it for you that you know the cause of your complaints?' some participants spontaneously started to describe risk factors like smoking. By changing the item into 'knowing what is wrong with you', this was solved.

Take a short break

The item 'can take a short break' gave many different interpretations, often without any relationship with the hospital. Several alternatives were tried: 'to recharge', 'to take a moment', but these did not improve the understanding. It was therefore decided to remove this item.

Remain alive

The item 'remain alive' gave mixed reactions. Some were irritated by the question. For others it was obvious that it was very important to them that they wanted to remain alive, by adding words like 'of course!'. However, there were also participants who deemed remaining alive less obvious and started to think about the question. For example:

> Item *remain alive*, step 1: *Yes, at least if my life stays well through it. But if it makes my life much worse, it doesn't matter for me any longer.* (*P6*)

Unless the mixed reactions to this question, it was remained because it was not obvious for all participants and because the researchers considered it unreasonable to have a questionnaire with many potential outcomes, but to omit the one outcome that for many participants is considered as the most important.

For peer review only

Additions

Participants gave the following suggestions which were added to the questionnaire: family life, driving, hobbies, urinating. The adaptations and additions led to P-BAS HOP Version 2.

General evaluation

Many participants stated that the questionnaire was quite easy to fill out, although this was not always congruent with the observations about their understanding. Several mentioned enjoying filling in the questionnaire. One participant mentioned that the tool was very important for him in order to state his own priorities. For another participant the questionnaire was considered emotional, because the questions were confronting and he was afraid that many goals were not feasible. For some the questionnaire was somewhat tiring.

Content Validity

The goals the participants mentioned in their own words, were qualified in the questionnaire as at least 'somewhat important' in almost all cases. For example:

> *Yes, that is the quality of life .. Yes, it is important that comes up to standard again. (..) Well, cycling that, that comes in second place. I think* ***walking*** *is more important than ehm .. (…) I have been a volunteer for more than forty years now, helping people fill out tax forms. I think that is important to me. And that is, that is, that is also the* ***volunteer work****. If it is somewhat possible I would like to do that again. (…) Ehm,* ***go on outings****. I would like to keep doing things like that. (...) I have two grandsons: one is 6 and one is 8. And they are with us then. Well, when they are on holiday, a day to an amusement park with those little boys. Very nice. But what is also nice, is a day with the boys to the petting zoo. Those children like that and I like it when they have fun. That's it. And those kind of things you hope to be able to do that normally again in the future. (P14)*

This participant filled in in the questionnaire: Walking: quite important, (volunteer) work: moderately important, go on outings: moderately important.

TSTI evaluation questionnaire

Sample evaluation questionnaire

Ten patients participated in the TSTI for the evaluation questionnaire at discharge. The sampling of the participants continued until the last version of the questionnaire was considered clear and did not reveal any new problems. Characteristics of the participants are displayed in the third column of Table 1.

For peer review only

Process of testing and adaptations

The process of testing and adapting the evaluation questionnaire was much faster, because many problems with layout and wording of individual items had already been solved in the baseline phase. In the first version, the wording appeared to be too complicated for some participants. For example:

> *P: I find the questions complicated. I: Yes, because what makes it complicated for you? P: Well it says: 'helped you, the hospitalisation helped you to ...' I always find that so difficult, that that is in there. Because what kind of answer should you give? (E5)*

Therefore, the original formulation: 'The hospitalisation helped me to….' Was changed into: 'Because of the hospitalisation….'. This adaptation was clear for all the following participants and led to Version 2.

4. Field test with Version 2. Item reduction based on mean impact score and correlation.

The Benefit Assessment Scale Version 2 consisted of 32 items. In the three month inclusion period, 492 consecutive eligible patients meeting the inclusion criteria were admitted on the selected wards. Of these patients, 238 were not approached for logistic reasons. Hence, 254 patients were approached for informed consent and 106 patients (42%) gave informed consent. Of the 106 included patients, the P-BAS was not administered 15 times because of lack of time (for example patient had to leave for treatment or discharge) or the patient was too tired. This resulted in 91 administered P-BAS questionnaires. Of the 91 participants, 20 answered the questionnaire independently written and 71 were interviewed by the research assistant. Characteristics of the participants are displayed in Table 2 and the results are shown in Table 3.

Table 2. Participants Field test (n=91)

Characteristic	n
Gender	
Male	63
Female	28
Age (years), median (range)	75 (70 – 96)
Native language	
Dutch	55
Local dialect	27
Frisian	3
Unknown	6

For peer review only

Educational level*	
Low	22
Middle	47
High	22
Specialty	
Medical	42
Surgical	23
Cardiology	26
Admission type	
Acute	60
Elective	31

* Definition educational level: Low= no education, primary school, basic vocational training; Middle = secondary education, vocational training; High = bachelor, master

Table 3. Scores of Version 2 Benefit Assessment Scale Baseline. (n=91)

				Importance				
	Missing		Does not apply to me now n (%)	Not at all n (%)	Some-what n (%)	Mode rately n (%)	Quite n (%)	Very n (%)
Item	Failed* n (%)	n.d.† n (%)						
Better	1 (1.1)	0	8 (8.8)	0	1 (1.1)	2 (2.2)	17 (18.7)	62 (68.1)
Weight	1 (1.1)	1 (1.1)	57 (62.6)	10 (11.0)	4 (4.4)	7 (7.7)	3 (3.3)	8 (8.8)
Condition	0	0	17 (18.7)	2 (2.2)	1 (1.1)	6 (6.6)	31 (34.1)	34 (37.4)
Energy	0	1 (1.1)	18 (19.8)	1 (1.1)	0	3 (3.3)	33 (36.3)	35 (38.5)
Pain	1 (1.1)	1 (1.1)	33 (36.3)	0	2 (2.2)	4 (4.4)	9 (9.9)	41 (45.1)
Bowel movements	0	1 (1.1)	58 (63.7)	4 (4.4)	2 (2.2)	1 (1.1)	12 (13.2)	13 (14.3)
Urinate	0	1 (1.1)	64 (70.3)	4 (4.6)	0	1 (1.1)	10 (11.0)	11 (12.1)
Shortness of breath	1 (1.1)	0	39 (42.9)	1 (1.1)	2 (2.3)	5 (5.5)	11 (12.1)	32 (35.2)
Walking	0	1 (1.1)	32 (35.2)	1 (1.1)	3 (3.3)	5 (5.5)	16 (17.6)	33 (36.3)
Moving	0	1 (1.1)	35 (38.5)	1 (1.1)	2 (2.2)	5 (5.5)	18 (19.8)	29 (31.9)
Appetite	0	2 (2.2)	55 (60.4)	1 (1.1)	2 (2.2)	6 (6.6)	9 (9.9)	16 (17.6)
Knowing what is wrong	0	1 (1.1)	32 (35.2)	2 (2.2)	2 (2.2)	1 (1.1)	12 (13.2)	41 (45.1)
Disease under control	0	1 (1.1)	10 (11.0)	0	0	2 (2.2)	15 (16.5)	63 (69.2)
Alive	0	1 (1.1)	2 (2.2)	2 (2.2)	1 (1.1)	1 (1.1)	13 (14.3)	71 (78.0)
Enjoy	0	2 (2.2)	20 (22.0)	0	0	2 (2.2)	13 (14.3)	54 (59.3)

For peer review only

Freedom	0	1 (1.1)	31 (34.1)	0	1 (1.1)	1 (1.1)	12 (13.2)	45 (49.5)
Cooking	0	2 (2.3)	51 (56.0)	1 (1.1)	4 (4.4)	2 (2.2)	14 (15.4)	17 (18.7)
Housework	0	1 (1.1)	51 (56.0)	2 (2.2)	5 (5.5)	5 (5.5)	10 (11.0)	17 (18.7)
Groceries	0	1 (1.1)	42 (46.2)	1 (1.1)	5 (5.5)	9 (9.9)	12 (13.2)	21 (23.1)
Wash and dress	0	2 (2.3)	51 (56.0)	0	0	2 (2.2)	14 (15.4)	22 (24.2)
Garden	0	1 (1.1)	48 (52.7)	2 (2.2)	1 (1.1)	8 (8.8)	10 (11.0)	21 (23.1)
Sports	0	1 (1.1)	46 (50.5)	7 (7.7)	2 (2.2)	9 (9.9)	7 (7.7)	19 (20.9)
Hobbies	0	1 (1.1)	39 (42.9)	1 (1.1)	2 (2.2)	4 (4.4)	13 (14.3)	31 (34.1)
Work	0	3 (3.3)	63 (69.2)	3 (3.3)	0	4 (4.4)	7 (7.7)	11 (12.1)
Driving	0	2 (2.2)	46 (50.2)	1 (1.1)	0	2 (2.2)	8 (8.8)	32 (35.2)
Outings	0	1 (1.1)	28 (30.8)	1 (1.1)	3 (3.3)	8 (8.8)	19 (20.9)	31 (34.1)
Visiting	0	3 (3.3)	28 (30.8)	1 (1.1)	4 (4.4)	6 (6.6)	19 (20.9)	30 (33.0)
Family life	0	4 (4.4)	39 (42.9)	0	0	2 (2.2)	12 (13.2)	34 (37.4)
Home	0	2 (2.3)	21 (23.1)	1 (1.1)	0	1 (1.1)	4 (4.4)	62 (68.1)
Independence	1 (1.1)	3 (3.3)	29 (31.9)	0	1 (1.1)	2 (2.2)	6 (6.6)	49 (53.8)

* Measurement failed: invalid answer due to two options filled in

† n.d. = not done. No answer was given.

As seen in Table 3, the number of missing values ranges from zero to four per item. The answer options with the lowest priorities were used the least, especially 'not at all important' and 'somewhat important'. Therefore, and also because the options 'somewhat' and 'moderately' were very close, we decided to remove the option 'moderately'.

Four dyads had a Spearman's rank-order correlation coefficient > 0.7: energy and condition (rs = .80); moving and walking (rs = .87); cooking and groceries (rs = .75); cooking and housekeeping (rs = .70) Therefore of these dyads, one item was removed (condition, moving and cooking), inspired by the information derived from the TSTI's.

Table 4 shows the items with mean impact scores, sorted per category and descending mean impact scores. From the categories with at least two items, the item with the lowest mean impact score was removed. To give participants still the opportunity to indicate their individual priorities, even when being a minority, we added an open option to add extra individual goals.

Table 4. Mean impact scores per category.

	**University hospital**			**Regional teaching hospital**		
**Goals**	**Applied (%)**	**Importance score (M)**	**Mean impact score**	**Applied (%)**	**Importance score (M)**	**Mean impact score**
**Remain alive**						
Remain alive	98	3.70	3.62	75	2.64	1.90
**Controlling disease**						
Controlling	89	3.76	3.34	29	2.43	0.70

For peer review only

disease						
**Improving condition**						
Feeling better	91	3.71	3.38	71	2.73	1.94
Energy	80	3.40	2.72	50	2.23	1.12
Condition	*81*	*3.27*	*2.66*	*65*	*2.34*	*1.53*
Weight	*36*	*1.84*	*0.66*	*9*	*2.33*	*0.20*
**Alleviating complaints**						
Pain	63	3.59	2.26	44	2.72	1.20
Breath	57	3.39	1.92	38	2.64	0.99
Appetite	38	3.09	1.18	35	2.39	0.83
Bowel	35	2.88	1.03	29	2.47	0.71
Urinate	*29*	*2.92*	*0.83*	*17*	*2.67*	*0.46*
**Enjoying life**						
Enjoying life	78	3.75	2.91	31	2.53	0.78
**Improving/maintaining social functioning**						
Outing	69	3.23	2.23	27	2.11	0.57
Visiting	68	3.22	2.20	21	1.91	0.40
Family life	*55*	*3.67*	*2.03*	*5*	*2.80*	*0.13*
**Knowing what is wrong**						
Wrong	64	3.52	2.27	39	2.58	0.99
**Regaining/maintaining independence, freedom**						
Home	76	3.85	2.94	15	2.50	0.39
Independence	67	3.78	2.52	17	2.44	0.42
Freedom	*66*	*3.71*	*2.43*	*23*	*2.54*	*0.59*
**Improving daily functioning**						
Walking	64	3.33	2.14	54	2.57	1.38
Moving	*61*	*3.31*	*2.02*	*34*	*2.54*	*0.86*
Driving	48	3.63	1.75	14	2.13	0.31
Groceries	53	2.98	1.59	19	2.30	0.44
Wash/dress	43	3.53	1.52	26	2.52	0.65
Cooking	*43*	*3.11*	*1.33*	*15*	*1.75*	*0.27*
Housework	*43*	*2.90*	*1.26*	*20*	*1.86*	*0.38*
**Resuming work/hobbies**						
Hobbies	57	3.39	1.92	20	2.00	0.40
Garden	47	3.12	1.46	16	1.24	0.20
Sports	49	2.66	1.30	23	1.58	0.37
Work	*28*	*2.92*	*0.83*	*13*	*1.92*	*0.24*

The removed items are indicated in italic.

Repetition field test in regional teaching hospital

The field test was repeated in a regional teaching hospital with the same items, but with fewer answer options and the questions in two steps, as explained in the next paragraph. In the eight week

For peer review only

inclusion period, 209 patients meeting the inclusion criteria were admitted on the wards. Of these patients, 56 were not approached for logistic reasons. 153 Were therefore approached for informed consent and 104 patients (67%) gave informed consent. The items with the lowest mean impact scores were the same for most categories, except for the categories independence/freedom, improving daily functioning and work/hobbies.

Splitting tool into two phases

Since some problems with understanding remained, especially the difficulties relating the goals to their own situation, as described in the TSTI, we decided to split the tool into two phases. In the first phase an inventory of subjects with problems or limitations was made. These could be problems/limitations at the moment of interview, at the moment of admission, or expected problems/limitations. In the second phase, only the importance was asked for the goals related to the subjects that applied. As this adaptation complicated the tool, we decided to use it as an interview-based tool. The item reduction and splitting into two phases, resulted in P-BAS HOP version 3.

5. TSTI with Version 3.

P-BAS HOP Version 3 was only suitable as an interview version, therefore, this interview was always conducted by an interviewer and an observant. Eight participants participated in the TSTI about Version 3. Characteristics of the participants are displayed in the last column of Table 1.

General understanding

In general, the tool in two phases was well-understood. For example:

> Item *shortness of breath*, phase 1, step 1: *No, I have no problems with that, you know, shortness of breath.* (*A1*)
>
> Or:
>
> Item *shortness of breath*, phase 1, step 1: *Yes, that is present! And for that reason, I am admitted here. My oxygen was too low. And my carbon dioxide level is not good, much too high. Yes, complication of, yes.* (*A3*)

We shortened the instructions, but did not modify the content of the tool. This last adaptation led to the final questionnaire (Appendix 2). The completion of this baseline questionnaire took 5 to 24 minutes, with a median of 11 minutes.

DISCUSSION

For peer review only

The Patient Benefit Assessment Scale for Hospitalised Older patients (P-BAS HOP) was constructed as a tool that should be capable both to identify the goals and priorities of the individual older hospitalised patient and to measure his relevant outcomes regarding hospitalisation.

The items of the P-BAS HOP were based on interviews with hospitalised older patients. Including patients in the generation of patient reported outcomes is not self-evident and is even absent in many cases.[17] But even when patients are involved in the generations of outcomes, they still only reflect the priorities of the overall patient population and not the individual patient. Therefore, the major advantage of the P-BAS HOP is that patients can indicate their individual priorities, which also leads to individual benefits.

Indicating individual priorities is also possible with the GAS, but the GAS is more time consuming, varying from 15-20 minutes for experienced assessors,[18] to 90 minutes per patient,[19] while the P-BAS HOP takes 5 to 24 minutes, with a median of 11 minutes.

The pilot and field tests of the P-BAS HOP started before we achieved complete saturation of the goals in the qualitative interviews. Therefore, patients had the possibility to add goals during the TSTI. Several goals were added during the TSTI, which also appeared later in the qualitative interviews.[14] Still, the qualitative interviews revealed later some extra target complaints, which were not included in the P-BAS HOP, such as vomiting, dizziness and sweating. Yet, in the final version of the P-BAS HOP, patients still have the opportunity to add personal goals which were not mentioned before.

As this is a formative tool, item reduction could be problematic since procedures suitable for reflective tools, such as based on factor analysis and internal consistency, are not relevant.[12] By using the mean impact score to reduce items, items considered least important by the overall sample were removed, though this does not take account of the priorities of individuals who deviate from the majority. For this reason the extra open option was added. Most removed items, based on mean impact score, were confirmed when repeated in the regional teaching hospital. The only exceptions were in the categories *improving daily functioning*, *resuming work/hobbies,* and *regaining/maintaining independence/freedom.*

In the categories improving daily functioning and resuming work/hobbies, the lowest priorities were 'housework' and 'work' in the first sample and 'driving' and 'gardening' in the second. Since driving and work were the second lowest priority in the second sample, the removal of housework and work could be justified.

In the category *regaining/maintaining independence/freedom* priorities in both hospitals were entirely opposite. We therefore have to conclude that we were too early to remove the item freedom. It is unclear whether these differences are caused by different contexts or because the field

For peer review only

test in the regional hospital was after splitting the questionnaire into two phases and therefore the questions were altered.

The P-BAS HOP is only tested in hospitalised patients without cognitive impairment. It is therefore unknown if it is suitable in other contexts and it might be too complex for patients with cognitive impairment. In addition, the P-BAS HOP is only tested in the Netherlands and the translated English version has not yet been tested. Therefore, it is unknown whether the P-BAS HOP is applicable in other languages and cultures.

The TSTI gave valuable insights into the understanding of the questionnaire and the completion behaviour of the participants. Many adaptations were made, but it proved difficult to make the questionnaire understandable for all patients. These kinds of difficulties were seen in various examples where the TSTI was used.[15, 20-22] Unfortunately, the final version is only suitable to be completed with an interviewer and not as a self-administered questionnaire. The TSTI gave a first indication of the content validity, but further quantitative research into the construct validity, reliability and responsivity is needed.

Conclusions

The Patient Benefit Assessment Scale for Hospitalised Older Patients (P-BAS HOP) is a potentially suitable interview-based tool to identify the priorities and relevant outcomes of the individual older hospitalised adult. Based on these data it is possible to compute an individual Patient Benefit Index, which is an overall value between 0 (no benefit) and 3 (maximal benefit), which reflects the achievement of the goals weighted by the importance. Further quantitative research is needed to investigate the construct validity, reliability and responsiveness.

Acknowledgements We would like to thank all patients who participated in the interviews for sharing their stories with us and their feedback on the different versions of the P-BAS HOP. We thank the panel members for their feedback on the draft version. We would also like to thank all the research assistants and students for the many interviews they conducted, especially Gerdine Boot for all the patients she interviewed in the regional teaching hospital. We would like to thank Daniël Bosold, Janet Vroomen, Caroline Mutsaers, Margaretha Schenkeveld and Anna Pot for their role in the translation process of the P-BAS HOP.

Funding This study was funded by an unrestricted grant from the Dutch Foundation for Effective Elderly Care and the University of Groningen.

Author's contribution MJvdK designed the study. MJvdK conducted the Three Step Test Interviews (TSTI's). MJvdK coded the data of the TSTI's. MJvdK and SEdR regularly discussed the codes and the progress and adaptations of the different versions of the P-BAS HOP. SEdR supervised the field test in

For peer review only

the University hospital, BCvM in the Regional teaching hospital. MJvdK wrote the first draft of the manuscript, GJD, BCvM and SEdR contributed significantly to subsequent manuscript revisions. All authors have read and approved the final version of the manuscript.

Competing interests None declared

Data sharing All data relevant to the study are included in the article

Patient consent All patients gave informed consent

Ethical approval

The Medical Ethics Research Committee of the UMCG (file number M16.192615 and M16.199647) confirmed that the Medical Research Involving Human Subjects Act did not apply to the research project. Official approval by the committee was therefore not required.

For peer review only

REFERENCES

1 Reuben DB, Tinetti ME. Goal-oriented patient care--an alternative health outcomes paradigm. *N Engl J Med* 2012;366:777-9 doi:10.1056/NEJMp1113631.

2 Tinetti ME, Naik AD, Dodson JA. Moving from disease-centered to patient goals-directed care for patients with multiple chronic conditions: Patient value-based care. *JAMA Cardiology* 2016;1:9-10 doi:10.1001/jamacardio.2015.0248.

3 Donnelly C, Carswell A. Individualized outcome measures: a review of the literature. *Can J Occup Ther* 2002;69:84-94.

4 Mangin D, Stephen G, Bismah V, et al. Making patient values visible in healthcare: a systematic review of tools to assess patient treatment priorities and preferences in the context of multimorbidity. *BMJ Open* 2016;6:e010903- doi:10.1136/bmjopen-2015-010903.

5 van Seben R, Reichardt L, Smorenburg S, et al. Goal-Setting Instruments in Geriatric Rehabilitation: A Systematic Review. *J Frailty Aging* 2017;6:37-45 doi:10.14283/jfa.2016.103.

6 Fried TR, Tinetti M, Agostini J, et al. Health outcome prioritization to elicit preferences of older persons with multiple health conditions. *Patient Educ Couns* 2011;83:278-82 doi:10.1016/j.pec.2010.04.032.

7 Stolee P, Awad M, Byrne K, et al. A multi-site study of the feasibility and clinical utility of Goal Attainment Scaling in geriatric day hospitals. *Disabil Rehabil* 2012;34:1716-26 doi:10.3109/09638288.2012.660600 [doi].

8 Battle CC, Imber SD, Hoehn-Saric R, et al. Target complaints as criteria of improvement. *Am J Psychother* 1966;20:184-92 doi:10.1176/appi.psychotherapy.1966.20.1.184 [doi].

9 Hesbacher PT, Rickels K, Weise C. Target symptoms: a promising improvement criterion in psychiatric drug research. *Arch Gen Psychiatry* 1968;18:595-600.

10 Schulman-Green DJ, Naik AD, Bradley EH, et al. Goal setting as a shared decision making strategy among clinicians and their older patients. *Patient Educ Couns* 2006;63:145-51 doi:S0738-3991(05)00275-2 [pii].

11 Augustin M, Reich C, Schaefer I, et al. Development and validation of a new instrument for the assessment of patient-defined benefit in the treatment of acne. *J Dtsch Dermatol Ges* 2008;6:113-20 doi:10.1111/j.1610-0387.2007.06540.x.

12 De Vet HCW, Terwee CB, Mokkink LB, et al. Measurement in Medicine. A Practical Guide. Cambridge: Cambridge University Press 2011.

13 Beaton DE, Bombardier C, Guillemin F, et al. Guidelines for the Process of Cross-Cultural Adaptation of Self-Report Measures. *Spine* 2000;25:3186-91 doi:10.1097/00007632-200012150-00014.

14 Van der Kluit MJ, Dijkstra GJ, de Rooij S,E. Goals of older hospitalised patients: a qualitative descriptive study. *BMJ open* 2019;9:e029993- doi:10.1136/bmjopen-2019-029993.

15 Pool JJM, Hiralal S, Ostelo, Raymond W J G, et al. Added value of qualitative studies in the development of health related patient reported outcomes such as the Pain Coping and Cognition List in patients with sub-acute neck pain. *Man Ther* 2010;15:43-7 doi:10.1016/j.math.2009.05.010.

16 Van Der Veer K, Ommundsen R, Hak T, et al. Meaning Shift of Items in Different Language Versions. A Cross-National Validation Study of the Illegal Aliens Scale. *Quality and Quantity* 2003;37:193-206.

17 Wiering B, de Boer D, Delnoij D. Patient involvement in the development of patient-reported outcome measures: a scoping review. *Health Expect* 2017;20:11-23 doi:10.1111/hex.12442 [doi].

18 Stolee P, Rockwood K, Fox RA, et al. The use of goal attainment scaling in a geriatric care setting. *J Am Geriatr Soc* 1992;40:574-8.

19 Toto PE, Skidmore ER, Terhorst L, et al. Goal Attainment Scaling (GAS) in geriatric primary care: a feasibility study. *Arch Gerontol Geriatr* 2015;60:16-21 doi:10.1016/j.archger.2014.10.022 [doi].

20 Jansen H, Hak T. The Productivity of the Three-step Test-interview (TSTI) Compared to an Expert Review of a Self-administered Questionnaire on Alcohol Consumption. *Journal of official statistics* 2005;21:103-20.

21 Liu RD, Buffart LM, Kersten MJ, et al. Psychometric properties of two physical activity questionnaires, the AQuAA and the PASE, in cancer patients. *BMC Med Res Methodol* 2011;11:30,2288-11-30 doi:10.1186/1471-2288-11-30 [doi].

22 van Alphen A, Westerman MJ, Visschedijk JH, et al. A qualitative study of Falls Efficacy Scale-International/Hips. What do we measure?. *Tijdschr Gerontol Geriatr* 2013;44:3-11 doi:10.1007/s12439-013-0005-9 [doi].

For peer review only

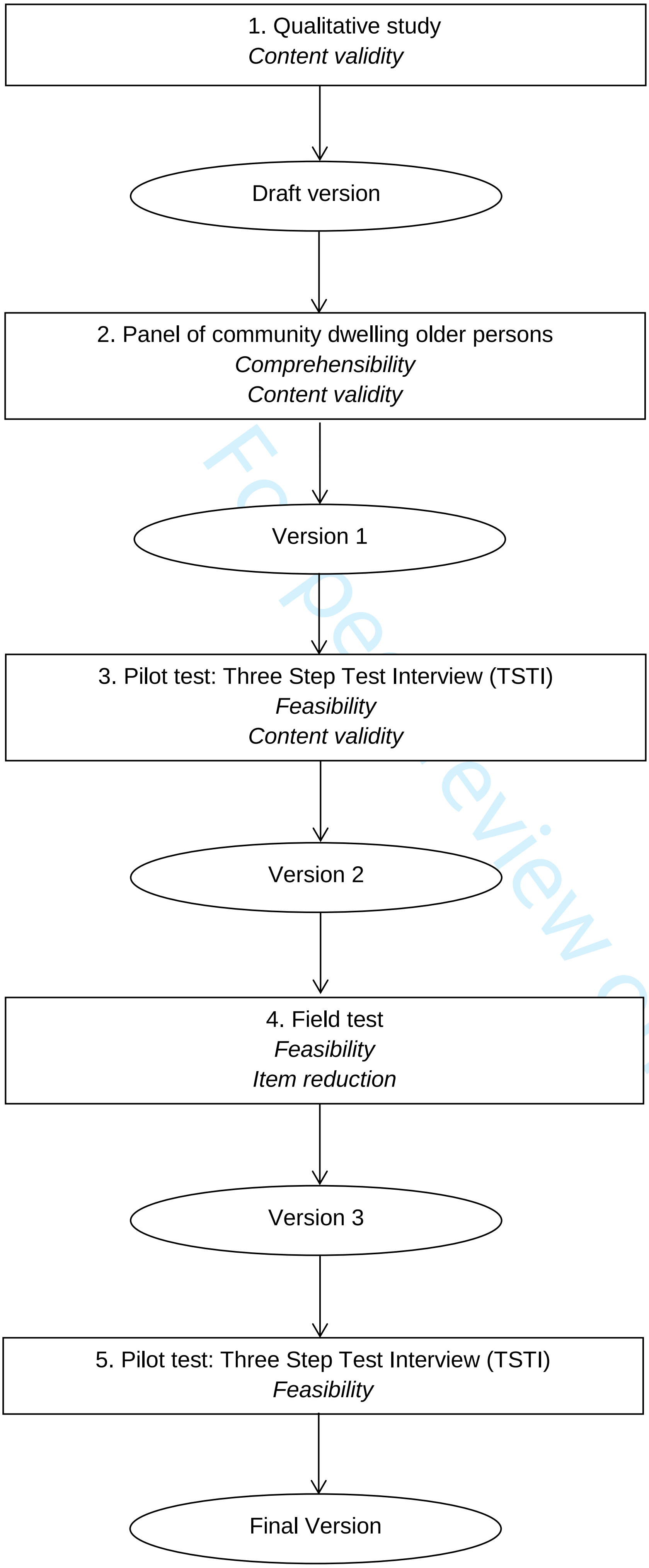

For peer review only

Appendix 1. Version 1. Patient Benefit Assessment Scale

Hospitalisation goals

The following questions cover how important the goals below are for you during your current hospitalisation.

Can you indicate how important each goal below is for you? You can choose from: ‘not at all’, ‘somewhat’, ‘moderately’ ‘quite’, or ‘very’. If a goal doesn't apply to you, for example because you don't have difficulty with the listed problem (for example with bowel movements, or shortness of breath) or because you don’t have a garden, then choose ‘does not apply to me’.

	How important is it for you that through this hospitalisation…	Not at all	Somewhat	Moderately	Quite	Very	Does not apply to me
1	You feel better						
2	You regain weight						
3	Your condition improves						
4	You have more energy						
5	You can walk better						
6	You move easier						
7	You can do housework						
8	You can cook						
9	You can do the groceries						
10	You can garden						
11	You can take pleasure in eating						
12	You can wash and dress yourself						
13	You can exercise or participate in sports						
14	You have no pain						
15	You have normal bowel movements						
16	You have less shortness of breath						
17	Your disease is under control						
18	You remain alive						
19	You can enjoy life						
20	You regain your freedom						
21	You can resume your (volunteer) work						
22	You can go on outings						
23	You can visit friends or family						
24	You can take a short break						
25	You know the cause of your complaints						
26	You can go back home						
27	You regain your independence						

For peer review only

Evaluation of hospitalisation goals

In the beginning of your hospitalisation you indicated how important various goals for you were. Can you indicate for each of the goals below how much the hospitalisation has helped to achieving the goal? You can indicate whether the hospitalisation helped you 'not at all', 'somewhat', 'moderately' 'quite', or 'very'. If a goal didn't apply to you, for example because you didn't have difficulty with the listed problem (for example with bowel movements, or shortness of breath) or because you have no garden, then choose 'did not apply to me'.

	The hospitalisation helped me to...	Not at all	Somewhat	Moderately	Quite	Very	Did not apply to me
1	Feel better						
2	Regain weight						
3	Improve my condition						
4	Have more energy						
5	Walk better						
6	Move easier						
7	Do the housework						
8	Cook						
9	Do the groceries						
10	Garden						
11	Take pleasure in eating						
12	Wash and dress myself						
13	Exercise or Participate in sports						
14	Have no pain						
15	Have normal bowel movements						
16	Have less shortness of breath						
17	Keep my disease under control						
18	Remain alive						
19	Enjoy life						
20	Regain my freedom						
21	Resume my volunteer work						
22	Go on outings						
23	Visit family or friends						
24	Can take a short break						
25	Know what the cause of my complaints is/was						
26	Go back to my home						
27	Regain my independence						

For peer review only

Appendix 2. Final Version Patient Benefit Assessment Scale

Hospitalisation Goals

People differ in what they wish to achieve with a hospitalisation. They have different goals. This depends on what they suffer from, what they are hospitalised for, and what they find important in life.

I am now going to mention some subjects that may be important to you during this hospitalisation. Can you say whether each applies to you?

A subject applies to you if you experience or anticipate problems or limitations and this applies to your life. For people who, for example, are short of breath, the subject shortness of breath probably applies, but not for others. For others they may be struggling to enjoy life because of their illness, but if you experience no problems with it, then this doesn't apply to you.

Instruction for the interviewer: Circle the number for the subject that applies to the participant.

If a participant asks what you mean by a subject, say:

Are you experiencing problems with [subject] now, or when you were admitted, or are you expecting problems with it because of your illness or hospitalisation?

For the functional subjects, prefer not to use the word 'problem', but rather 'trouble' or 'limitation'.

Now follow the subjects, you should indicate whether each subject applies to you or not:

1. Wanting to feel better

Explanation if necessary: do you feel sick or miserable and would you like to feel better?

2. Energy
3. Pain
4. Bowel movements
5. Shortness of breath
6. Walking
7. Appetite
8. Unclarity about what is wrong with me

Explanation if necessary: you may be wondering what is wrong with you. If this was already clear for you when you were admitted then this does not apply.

9. Controlling my disease

Explanation if necessary: Do you suffer from a disease that needs to be controlled?

10. Remain alive

Explanation if necessary: Was your life in danger when you were admitted or do you think that this hospitalisation must contribute to remain alive?

11. Enjoying life
12. Housework
13. Groceries
14. Washing and dressing myself
15. Gardening
16. Exercise or Sports
17. Hobbies
18. Driving
19. Going on outings
20. Visiting family or friends

For peer review only

21. Return back to my home
Explanation if necessary: Are you unsure whether you can return to your own home?
22. Independence
23. Are there other themes which are important to you during this hospitalisation that I have not mentioned yet? If so, which?

Now that we have identified the themes that apply to you, I am going to ask to what extent they are goals during this hospitalisation.

Can you indicate for each goal that I mention how important it is during this hospitalisation? You can choose from 'not at all important', 'moderately important' 'quite important', or 'very important'.

For some goals you may still say that they do not apply, for example because they do not matter during this hospitalisation. Then you indicate "does not apply to me now".

Instruction to interviewer: Read only the goals which you have circled.

For peer review only

		does **not apply** to me now	**not at all** important	**somewhat** important	**quite** important	**very** important
1	How important is it for you that you **feel better again** as a result of this hospitalisation	☐	☐	☐	☐	☐
2	How important is it for you that you **have more energy** as a result of this hospitalisation	☐	☐	☐	☐	☐
3	How important is it for you that you **have less pain** as a result of this hospitalisation	☐	☐	☐	☐	☐
4	How important is it for you that you **have normal bowel movements again** as a result of this hospitalisation	☐	☐	☐	☐	☐
5	How important is it for you that you **are less short of breath** as a result of this hospitalisation	☐	☐	☐	☐	☐
6	How important is it for you that you can **walk better again** as a result of this hospitalisation	☐	☐	☐	☐	☐
7	How important is it for you that you **regain your appetite** as a result of this hospitalisation	☐	☐	☐	☐	☐
8	How important is it for you that you **know what is wrong with you** as a result of this hospitalisation	☐	☐	☐	☐	☐
9	How important is it for you that **your disease is under control** as a result of this hospitalisation	☐	☐	☐	☐	☐
10	How important is it for you that you **remain alive** as a result of this hospitalisation	☐	☐	☐	☐	☐
11	How important is it for you that you can **enjoy life again** as a result of this hospitalisation	☐	☐	☐	☐	☐
12	How important is it for you that you can **do housework again** as a result of this hospitalisation	☐	☐	☐	☐	☐

		does **not apply** to me now	**not at all** important	**somewhat** important	**quite** important	**very** important
13	How important is it for you that you can **do the groceries again** as a result of this hospitalisation	☐	☐	☐	☐	☐
14	How important is it for you that you **can wash and dress yourself again** as a result of this hospitalisation	☐	☐	☐	☐	☐
15	How important is it for you that you **can garden again** as a result of this hospitalisation	☐	☐	☐	☐	☐
16	How important is it for you that you **can exercise or participate in sports again** as a result of this hospitalisation	☐	☐	☐	☐	☐
17	How important is it for you that you **can exercise your hobbies again** as a result of this hospitalisation	☐	☐	☐	☐	☐
18	How important is it for you that you **can drive again** as a result of this hospitalisation	☐	☐	☐	☐	☐
19	How important is it for you that you **go on outings again** as a result of this hospitalisation	☐	☐	☐	☐	☐
20	How important is it for you that you **can visit family or friends again** as a result of this hospitalisation	☐	☐	☐	☐	☐
21	How important is it for you that you **can return to your own home again** as a result of this hospitalisation	☐	☐	☐	☐	☐
22	How important is it for you that you **regain your independence** as a result of this hospitalisation	☐	☐	☐	☐	☐
23	How important is it for you that you ____________________ as a result of this hospitalisation	☐	☐	☐	☐	☐
24	Do you have any goals I haven't mentioned yet? If so: How important is it to you that____________________________ as a result of this hospitalisation	☐	☐	☐	☐	☐

Evaluation of hospitalisation goals

In the beginning of your hospitalisation you indicated how important various goals for you were. A goal is something you want to achieve with a hospitalisation. Some goals you may have achieved, others maybe not or not entirely.

Can you indicate for each of the goals below how much the hospitalisation has helped to achieving the goal? You can indicate whether the hospitalisation has helped you 'not at all', 'moderately', 'quite', or 'completely'.

Only the goals which applied at baseline are evaluated with the participant.

		Not at all	Somewhat	Quite	Completely
1	Because of the hospitalisation I **feel better again**	☐	☐	☐	☐
2	Because of the hospitalisation I have **more energy**	☐	☐	☐	☐
3	Because of the hospitalisation I have **no more pain**	☐	☐	☐	☐
4	Because of the hospitalisation I have **normal bowel movements again**	☐	☐	☐	☐
5	Because of the hospitalisation I am **less short of breath**	☐	☐	☐	☐
6	Because of the hospitalisation I **walk better again**	☐	☐	☐	☐
7	Because of the hospitalisation I **regained appetite**	☐	☐	☐	☐
8	Because of the hospitalisation I **know what is/ was wrong with me**	☐	☐	☐	☐
9	Because of the hospitalisation **my disease is under control**	☐	☐	☐	☐
10	Because of the hospitalisation I **remained alive**	☐	☐	☐	☐
11	Because of the hospitalisation I **enjoy life again**	☐	☐	☐	☐
12	Because of the hospitalisation I **do my housework again**	☐	☐	☐	☐
13	Because of the hospitalisation I **do the groceries again**	☐	☐	☐	☐
14	Because of the hospitalisation I **wash and dress myself again**	☐	☐	☐	☐
15	Because of the hospitalisation I **garden again**	☐	☐	☐	☐
16	Because of the hospitalisation I **participate in sports again**	☐	☐	☐	☐

		Not at all	Somewhat	Quite	Completely
17	Because of the hospitalisation I **can resumed my hobbies**	☐	☐	☐	☐
18	Because of the hospitalisation I **drive again**	☐	☐	☐	☐
19	Because of the hospitalisation I **go on outings again**	☐	☐	☐	☐
20	Because of the hospitalisation I **visit family or friends again**	☐	☐	☐	☐
21	Because of the hospitalisation I **am back in my own home**	☐	☐	☐	☐
22	Because of the hospitalisation I **regained independence**	☐	☐	☐	☐
23	Because of the hospitalisation ___________________________	☐	☐	☐	☐

For peer review only

BMJ Open

Development of a new tool for the assessment of patient defined benefit in hospitalised older patients: the Patient Benefit Assessment Scale for Hospitalised Older Patients (P-BAS HOP)

Journal:	*BMJ Open*
Manuscript ID	bmjopen-2020-038203.R1
Article Type:	Original research
Date Submitted by the Author:	07-Jul-2020
Complete List of Authors:	van der Kluit, Maria; University of Groningen, University Medical Center Groningen, University Center for Geriatric Medicine<br>Dijkstra, Geke; Department of Health Sciences, Applied Health Research<br>van Munster, Barbara; University of Groningen, University Medical Center Groningen, University Center for Geriatric Medicine; Gelre Hospitals, Geriatrics<br>De Rooij, Sophia; University Medical Center Groningen, University Center for Geriatric Medicine; Medical Spectrum Twente, Medical School Twente
**Primary Subject Heading**:	Geriatric medicine
Secondary Subject Heading:	Communication, Health services research, Research methods
Keywords:	GERIATRIC MEDICINE, QUALITATIVE RESEARCH, Quality in health care < HEALTH SERVICES ADMINISTRATION & MANAGEMENT

SCHOLARONE™
Manuscripts

I, the Submitting Author has the right to grant and does grant on behalf of all authors of the Work (as defined in the below author licence), an exclusive licence and/or a non-exclusive licence for contributions from authors who are: i) UK Crown employees; ii) where BMJ has agreed a CC-BY licence shall apply, and/or iii) in accordance with the terms applicable for US Federal Government officers or employees acting as part of their official duties; on a worldwide, perpetual, irrevocable, royalty-free basis to BMJ Publishing Group Ltd ("BMJ") its licensees and where the relevant Journal is co-owned by BMJ to the co-owners of the Journal, to publish the Work in this journal and any other BMJ products and to exploit all rights, as set out in our licence.

The Submitting Author accepts and understands that any supply made under these terms is made by BMJ to the Submitting Author unless you are acting as an employee on behalf of your employer or a postgraduate student of an affiliated institution which is paying any applicable article publishing charge ("APC") for Open Access articles. Where the Submitting Author wishes to make the Work available on an Open Access basis (and intends to pay the relevant APC), the terms of reuse of such Open Access shall be governed by a Creative Commons licence – details of these licences and which Creative Commons licence will apply to this Work are set out in our licence referred to above.

Other than as permitted in any relevant BMJ Author's Self Archiving Policies, I confirm this Work has not been accepted for publication elsewhere, is not being considered for publication elsewhere and does not duplicate material already published. I confirm all authors consent to publication of this Work and authorise the granting of this licence.

For peer review only

Development of a new tool for the assessment of patient defined benefit in hospitalised older patients: the Patient Benefit Assessment Scale for Hospitalised Older Patients (P-BAS HOP)

Authors:

1. Maria Johanna van der Kluit, MSc RN (Corresponding author)

   University of Groningen, University Medical Center Groningen, University Center for Geriatric Medicine, Hanzeplein 1, 9700 RB Groningen, The Netherlands

   m.j.van.der.kluit@umcg.nl

   +31503613921

2. Geke J. Dijkstra, PhD

   University of Groningen, University Medical Center Groningen, Department of Health Sciences, Applied Health Research, Groningen, The Netherlands

   g.j.dijkstra@umcg.nl

3. Barbara C. van Munster, MD PhD

   University of Groningen, University Medical Center Groningen, University Center for Geriatric Medicine, Groningen, The Netherlands.

   Department of Geriatrics, Gelre Hospitals, Apeldoorn, The Netherlands.

   b.c.van.munster@umcg.nl

4. Sophia E. de Rooij, MD PhD

   University of Groningen, University Medical Center Groningen, University Center for Geriatric Medicine, Groningen, The Netherlands

   Medical Spectrum Twente, Medical School Twente, Enschede, The Netherlands

   sejaderooij@gmail.com

Words: 4692

Words abstract: 299

References: 26

Figures: 1

Tables: 4

For peer review only

ABSTRACT

Objectives: To support the shift from disease-oriented towards goal-oriented care, we aimed to develop a tool which is capable both to identify priorities of an individual older hospitalised patient and to measure the outcomes relevant to him.

Design: Mixed methods design with open interviews, Three Step Test Interviews (TSTI), and a quantitative field test.

Setting: University teaching hospital and a regional teaching hospital.

Participants: Hospitalised patients ages 70 years and older.

Results: The Patient Benefit Assessment Scale for Hospitalised Older Patients (P-BAS HOP) consists of a baseline questionnaire and an evaluation questionnaire. Items were based on 15 qualitative interviews with hospitalised older patients. Feedback from a panel of four community-dwelling older persons resulted in some adaptations to wording and one additional item. Twenty-six hospitalised older patients participated in TSTIs with Version 1 of the baseline questionnaire, revealing indications for a good content validity and barriers in completion behaviour, global understanding, and understanding of individual items, which were solved with several adaptations. Four additions were made by participants. After TSTIs with ten patients with the evaluation questionnaire, one adaptation was made. A field test with 91 hospitalised older patients revealed a small number of missing values.

To enhance the feasibility, the number of items was reduced from 32 to 23, based on correlations and mean impact score. The field test was repeated with 104 other patients in a regional teaching hospital. To enhance the understanding, the tool was split into two phases. This version was tested with TSTIs with eight patients and appeared to be understandable. The final version was an interview-based tool and took about 11 minutes to complete.

Conclusions: The P-BAS HOP is a potentially suitable tool to identify priorities and relevant outcomes of the individual patient. Further research is needed to investigate its validity, reliability and responsiveness.

Keywords Geriatric medicine; Older adults; Hospitalisation; Patient perspective; Goal setting; Patient-reported outcomes; Tool; Value-Based Health Care

ARTICLE SUMMARY

Strengths and limitations of this study

- The content of the P-BAS HOP is based on open interviews with hospitalised older patients.
- Patients are able to indicate their individual outcome priorities.

For peer review only

- The P-BAS HOP is tested intensively in the target population with Three Step Test Interviews. This gave valuable insights into the understanding of the tool and the completion behaviour of the participants.
- The current version of the P-BAS HOP is only suitable to be completed with an interviewer and not as a self-administered questionnaire.
- It is unknown whether the P-BAS HOP is feasible in other healthcare systems, languages and cultures than in the Netherlands.

For peer review only

BACKGROUND

To fit the needs of the ageing population, and patients with multiple chronic diseases, a shift is recommended from disease-oriented towards goal-oriented care. Older patients with multimorbidity may be more interested in more personal goals such as for them important symptoms, functional status and social functioning than in traditional outcomes such as survival and biomarkers,[1, 2] but these goals and outcomes differ per individual.[3, 4] When care would be systematically evaluated by personal goal-oriented outcomes, a tool is needed which is capable both to identify the priorities of the individual patient and to measure the outcomes relevant to him.

Three literature reviews[5-7] into tools used to assess patient outcome priorities in the context of multimorbidity revealed a few potentially useful tools. Tools only suitable for specific activities, such as the Canadian Occupational Performance Measure (COPM),[5, 7] Self-Identified Goals Assessment (SIGA),[7] Assessment of Motor and Process Skills (AMPS) and McMaster Toronto Arthritis (MACTAR)[5] were ignored. A general tool is the Outcome Prioritization Instrument,[6, 8] which is suitable to elicit four patient priorities, but these priorities are still very global and it remains unclear how to evaluate them after treatment. Another tool is the Goal Attainment Scaling (GAS)[5, 7] which is designed to set and evaluate individualized goals and outcomes. Disadvantages of the GAS are that it can be too challenging for patients to articulate their own goals and that it is time consuming.[9] The International Classification of Functioning, Disability and Health (ICF) framework for goal setting is used to categorize patient goals set in semi-structured interviews, but has still the same disadvantages as the GAS and has, in addition, a very poor responsiveness.[7] Finally, with the Target Complaints,[5] the patient defines target complaints as those problems for which help was sought. These complaints are scored at the beginning and at the end of the treatment by the patient, or the patient rates the degree of improvement.[10, 11] The Target Complaints is individualized and patient-centred. However, it focuses solely on problems and not on goals. Disadvantages for the GAS, ICF and Target Complaints could be that for some older patients it might be difficult to formulate their own goals and problems because many older persons are not accustomed to defining and discussing personal goals and prompting is often necessary.[12] The quality of the answers is therefore dependent on the interviewer's experiences and techniques.

For this reason, another method of defining patient-defined goals and outcomes was sought and found in the literature about treatment of acne. Augustin et al.[13] developed a tool consisting of two parts: 1) a baseline questionnaire to assess the importance of various predefined goals, based on themes derived from qualitative interviews in patients with acne and 2) an evaluation questionnaire to evaluate the extent to which treatment helped to achieve these goals. Based on these data it is possible to compute an individual Patient Benefit Index. This is an overall value between 0 (no benefit) and 4 (maximal benefit), which reflects the achievement of the goals weighted by the

For peer review only

importance.[13] The advantage of this tool is the insight into the individualized patient perspective, together with standardisation.

The aim of this study was to develop a tool to inventory individual goals and benefits of older hospitalised patients, based on the model of Augustin et al.[13] This article presents its development, early testing and adaptations.

METHODS

The steps used to develop the Patient Benefit Assessment Scale for Hospitalised Older Patients (P-BAS HOP) are based on the steps of De Vet et al.[14] and outlined in Figure 1. After each step, the tool was adapted. The steps are explained in the following sections. For the readability, the methods and results of each step are alternated. The P-BAS was developed and tested in Dutch. The P-BAS was translated into English in a translation - back translation procedure involving four translators (two native English, two native Dutch), a language professional and authors MJvdK and GD.[15]

Patient and public involvement

Patients and public were involved in the generation of the items, the importance and relevance of the items and the assessment of the feasibility and understanding of the tool.

Figure 1. Development of the P-BAS HOP

1. Qualitative study

Firstly, open interviews with hospitalised older medical and surgical patients about their goals regarding their hospitalisation were performed. The description of these goals is published elsewhere.[3] These goals were then coded inductively and transformed into questionnaire items, and the first draft of the P-BAS HOP was then constructed, consisting of a baseline questionnaire and an evaluation questionnaire.

2. Panel of community-dwelling older persons

The first draft version was proposed by e-mail to a panel of community-dwelling older persons to assess the comprehensibility and relevance of the items and the tool and ask for omissions or redundancies.

Results

Four community-dwelling older persons gave written feedback on the draft version of the tool. This led to adaptation in wording of the introduction text and to some items which were not clear enough

For peer review only

or could be interpreted in multiple ways. An example of an item that was adapted: for the item ‘to be able to eat’, it was not clear if it concerned the instrumental activity of eating or concerned appetite. Therefore, the item was changed into ‘can take pleasure in eating’.

Further, one additional item was added, namely ‘to wash and dress yourself’ and the sequence of two items was changed. Version 1 of the P-BAS HOP is shown in Appendix 1.

3. Pilot test: Three Step Test Interview (TSTI).

The adapted tool (Version 1, Appendix 1) was tested with the TSTI[16, 17] in older hospitalised patients. The TSTI is a type of cognitive interview suitable to assess how people interpret a questionnaire, its different items and what kind of strategies they use in responding to them. The TSTI consists of the following steps:

Step 1: Concurrent thinking aloud

The participant completed version 1 of the P-BAS HOP while thinking aloud. The interviewer observed, made notes of the participant’s behaviour (hesitations, skipping questions, corrections) and verbalized thoughts. However, the interviewer did not talk, or intervene. The instructions for the participant were: Please fill in this questionnaire and try to think aloud about what your thoughts are while reading the questions and choosing the right response category.

Step 2: Retrospective interview

With the retrospective interview any gaps from the first step were filled in. Every behaviour and thought from the observation of which the interviewer wanted further information, was clarified.

Step 3: Semi-structured interview

An in-depth interview was conducted, aimed at eliciting the participant’s considerations and opinions. The participant was given the opportunity to explain behaviour, actions or thoughts that he had in the previous steps. The participant was asked how he understood different items, was asked for any omissions and his opinion about filling in the questionnaire. The participant was also asked to explain his goals in his own words in order to perform a first content validation of the P-BAS HOP.

Participants

Eligible participants of the TSTI were 70 years and older; planned or unplanned hospitalised on medical or surgical wards of a university teaching hospital in the Netherlands; able to speak and understand Dutch and were without cognitive impairment. Inclusion criteria were verified with the staff nurse, and patients were then approached by the interviewer (MJvdK). Participants were completely anonymous, no list with names or other identifying data was made, nor did the researchers have access to medical records. Participants gave verbal consent to the interview and audio recording.

For peer review only - http://bmjopen.bmj.com/site/about/guidelines.xhtml

Data analysis

Data gathering and data analysis were alternated. Interviews were audio recorded and transcribed verbatim. All remarks were then organised by question and step. After that, the data were coded by MJvdK and grouped into categories. The tool was adapted several times after the feedback until it was considered feasible and understandable.

The TSTI was repeated with the evaluation questionnaire. This was done at patient discharge.

Results

Sample baseline questionnaire

Twenty-six older hospitalised patients participated in the TSTI. Characteristics of the participants are displayed in the second column of Table 1.

Table 1. Participants Three Steps Test Interview (TSTI) baseline questionnaire version 1, evaluation questionnaire version 1 and baseline questionnaire version 3.

	Baseline questionnaire version 1 (n=26)	Evaluation questionnaire version 1 (n=10)	Baseline questionnaire version 3 (n=8)
Characteristic	n	n	n
Gender			
Male	19	8	4
Female	7	2	4
Age (years)			
70-79	18	8	7
80-89	7	2	0
90-99	1	0	1
Native language			
Dutch	14	7	7
Local dialect	10	2	1
Frisian	2	0	0
Foreign language	0	1	0
Educational level*			
Low	8	5	2

For peer review only

Middle	10	4	5
High	8	1	1
Admission reason†			
Cardiac problems	9	1	1
Pulmonary problems	7	3	2
Bowel problems	2	2	1
Fever/ infection	2	2	0
Vascular surgery	2	1	0
Cancer	2	0	0
Accident/fracture(s)	2	0	1
Kidney problems	0	1	1
Syncope	0	0	1
Ulcera	0	0	1

* Definition educational level: Low= no education, primary school, basic vocational training; Middle = secondary education, vocational training; High = bachelor, master

† Reason according to the patient

For peer review only

Coding categories

The codes were sorted into the following categories: Completion behaviour, Global understanding, Understanding and reactions regarding individual items, Additions, General evaluation, and Content validity.

Completion behaviour

With 'completion behaviour' is meant the behaviour participants showed when completing the questionnaire. Observations revealed that many participants skipped the instruction text partly or even completely. For some participants, reading these instructions was demanding, others did not understand how a table works and where to place a mark. Adding an example table with instructions showing how and where to place a mark brought no improvements. In the final version boxes to tick were included in the table.

Global understanding

Many examples of correct understanding were coded. For example:

> Item *regaining weight*, step 1: *Oops, dear, pooh, let's see, that is certainly important, because I have lost weight lately since I had not been feeling well for a while, that is, not*

shortness of breath or anything, but, very tired, listless. So, a little weight gain is important. (P9)

Or:

Item *walking*, step 1: *Well, I walk well. Doesn't apply to me.* (*P17*)

A few participants interpreted the questions as if it was an evaluation of their current level of functioning. For example:

Item *energy*, step 2: *I: You have filled in 'not at all' in 'you have more energy'. What is the reason that you just ...? P: Because I feel lethargic. That is what I mean to say. I used to be a very energetic person. (…) That is gone. (…) That is what I mean by that question. I: Yes, so you actually filled in how you are feeling now. P: Yes, now. At the time. I: Yes, so you say .. P: Not from last year or half a year ago. They are snapshots, aren't they? That was what you meant, right? (...) I: And when I ask you the question: 'How important is it that you get energy again?' P: Very important. (…) Because I've always been energetic. Very important.* (*P2*)

Other participants had difficulties relating the goals to their own situation. For example:

Item *shortness of breath*, step *1 I'm actually never short of breath. But it is quite important.* (*P25*)

There were also participants who did recognize that a certain goal did not apply for them, but they did not understand how to indicate that in the tool.

Some participants were reluctant to use the options 'not at all important' or 'doesn't apply to me', because they deemed those answers socially undesirable.

Since many older persons have multiple health problems, it is possible that a participant experiences a problem with an item, but is admitted for another health problem. Many participants were able to make this distinction. For example:

Item *moving*, step 1: *Yes, that will never be all right again, I can tell. Does no longer apply at all. Already 30 years ago they said: Mr. B., you have to learn to live with that. And they still say that today. Osteoarthritis, there is nothing you can do about it.* (*P18*)

But for others this distinction was more difficult.

Adaptations

To enhance the general understanding, the following adaptations to the tool were made and tested in new participants:

Several adaptations were made in the instruction text.

For peer review only

In the columns with the answer options the word ‘important’ was added to all answer options. For example: ‘very’ was changed into ‘very important’, to make clear that the question was not to evaluate current function, but to indicate how important the goal was.

The sequence of the questions was changed. To enable participants to relate the goals to their own situation, the questions related to somatic complaints were moved to the beginning of the questionnaire.

Another adaptation made to improve the understanding to relate the goals to the patients’ own situation, was to add the word ‘again’ to the goals, to make clear that it is something they had before and they have to regain by the hospital admission. For example: *How important is it to you that you have normal bowel movements again*.

The next adaptation was to move the answer option ‘doesn’t apply to me’ from the last to the first column. This made it easier to find that option.

The final adaptation to improve making the connection between the hospital admission and their goals, was repeating the question in every line. Instead of having the text ‘*How important is it to you that by this hospital admission…’* on top of the page alone, this question was repeated in every row. Apart from this, several adaptations were made to the layout in order to ease the reading for participants.

Understanding and reactions regarding individual items

The following individual items caused discussion: take pleasure in eating, to know the cause of your complaints, take a short break, and remain alive.

Take pleasure in eating

Some participants had a more epicurean association with this item. Therefore, it was changed into: ‘regain appetite’.

Cause of complaints

With the item ‘how important is it for you that you know the cause of your complaints?’ some participants spontaneously started to describe risk factors like smoking. By changing the item into ‘knowing what is wrong with you’, this was solved.

Take a short break

The item ‘can take a short break’ gave many different interpretations, often without any relationship with the hospital. Several alternatives were tried: ‘to recharge’, ‘to take a moment’, but these did not improve the understanding. It was therefore decided to remove this item.

For peer review only

Remain alive

The item 'remain alive' gave mixed reactions. Some were irritated by the question. For others it was obvious that it was very important to them that they wanted to remain alive, by adding words like 'of course!'. However, there were also participants who deemed remaining alive less obvious and started to think about the question. Unless the mixed reactions to this question, it was remained because it was not obvious for all participants and because the researchers considered it unreasonable to have a questionnaire with many potential outcomes, but to omit the one outcome that for many participants is considered as the most important.

Additions

Participants gave the following suggestions which were added to the questionnaire: family life, driving, hobbies, urinating. The adaptations and additions led to P-BAS HOP Version 2.

General evaluation

Many participants stated that the questionnaire was quite easy to fill out, although this was not always congruent with the observations about their understanding. Several mentioned enjoying filling in the questionnaire. One participant mentioned that the tool was very important for him in order to state his own priorities. For another participant the questionnaire was considered emotional, because the questions were confronting and he was afraid that many goals were not feasible. For some the questionnaire was somewhat tiring.

Content Validity

The goals the participants mentioned in their own words, were qualified in the questionnaire as at least 'somewhat important' in almost all cases. For example:

> *Yes, that is the quality of life .. Yes, it is important that comes up to standard again. (..) Well, cycling that, that comes in second place. I think* ***walking*** *is more important than ehm .. (…) I have been a volunteer for more than forty years now, helping people fill out tax forms. I think that is important to me. And that is, that is, that is also the* ***volunteer work****. If it is somewhat possible I would like to do that again. (…) Ehm,* ***go on outings****. I would like to keep doing things like that. (P14)*

This participant filled in in the questionnaire: Walking: quite important, (volunteer) work: moderately important, go on outings: moderately important.

TSTI evaluation questionnaire

For peer review only

Sample evaluation questionnaire

Ten patients participated in the TSTI for the evaluation questionnaire at discharge. The sampling of the participants continued until the last version of the questionnaire was considered clear and did not reveal any new problems. Characteristics of the participants are displayed in the third column of Table 1.

Process of testing and adaptations

The process of testing and adapting the evaluation questionnaire was much faster, because many problems with layout and wording of individual items had already been solved in the baseline phase. In the first version, the wording appeared to be too complicated for some participants. Therefore, the original formulation: 'The hospitalisation helped me to….' Was changed into: 'Because of the hospitalisation….'. This adaptation was clear for all the following participants and led to Version 2.

4. Field test with Version 2. Item reduction based on mean impact score and correlation.

Version 2 was tested with a new group of hospitalised older patients. The aim of this field test was to assess the feasibility of the P-BAS HOP in combination with other questionnaires. The trained research assistants observed during the field test that the tool was too time consuming and that some patients still had difficulties relating the questions to their personal situation, as was observed in the TSTI. Therefore, the following extra adaptations were made: item reduction, answer option reduction, and splitting the tool into two phases.

Participants

Eligible participants were consecutive patients aged 70 years and older; planned or unplanned hospitalised on medical or surgical wards of a university teaching hospital, expected to stay for at least 48 hours; and at maximal four days hospitalised at the moment of interviewing; able to speak and understand Dutch and were without cognitive impairment. Inclusion criteria were verified with the staff nurse. Patients were approached by a trained research assistant and gave signed informed consent to participate. The questionnaire was then conducted in a face to face interview with the research assistant, but to patients in a better condition and with middle or higher education the opportunity was given to fill in the questionnaire themselves, an option which only a minority of patients choose.

Item reduction

For peer review only

As this is a formative tool, item reduction procedures suitable for reflective tools, such as based on factor analysis and Cronbach's alpha, are not relevant.[14] Item reduction was therefore based on correlation and mean impact score.

Items within one category with a strong correlation, measured probably the same construct. Therefore, from dyads with a Spearman's rank-order correlation > 0.7, one item was removed.[14] For the calculation of the Spearman's rank-order correlation coefficient, the answer option 'does not apply to me now' and 'not at all important' were coded as 0, the options somewhat, moderately, quite important and very important were coded respectively as 1, 2, 3, 4.

For the reduction based on mean impact score, all items were sorted into categories. For each item the mean impact score was calculated: [% for whom the item played a role] * [mean importance for that item]. From every category with two or more items, the item with the lowest mean impact score was removed.[14] The field test was repeated in a regional teaching hospital by a trained research assistant, to check whether the impact differed in another context.

Results

The Benefit Assessment Scale Version 2 consisted of 32 items. In the three month inclusion period, 492 consecutive eligible patients meeting the inclusion criteria were admitted on the selected wards. Of these patients, 238 were not approached for logistic reasons, for example the patient could not be interviewed within the first four days because of absence for treatment, transfer from ward, shortage of research assistants. Hence, 254 patients were approached for informed consent and 106 patients (42%) gave informed consent. Of the 106 included patients, the P-BAS was not administered 15 times because of lack of time (for example patient had to leave for treatment or discharge) or the patient was too tired. This resulted in 91 administered P-BAS questionnaires. Of the 91 participants, 20 answered the questionnaire independently written and 71 were interviewed by the research assistant. Characteristics of the participants are displayed in Table 2 and the results are shown in Table 3.

Table 2. Participants Field test (n=91)

Characteristic	n
Gender	
Male	63
Female	28
Age (years), median (range)	75 (70 – 96)
Native language	

For peer review only

Dutch	55
Local dialect	27
Frisian	3
Unknown	6
Educational level*	
Low	22
Middle	47
High	22
Specialty	
Medical	42
Surgical	23
Cardiology	26
Admission type	
Acute	60
Elective	31

* Definition educational level: Low= no education, primary school, basic vocational training; Middle = secondary education, vocational training; High = bachelor, master

Table 3. Scores of Version 2 Benefit Assessment Scale Baseline. (n=91)

				Importance				
	**Missing**		**Does not apply to me now n (%)**	**Not at all n (%)**	**Some-what n (%)**	**Moderately n (%)**	**Quite n (%)**	**Very n (%)**
**Item**	**Failed* n (%)**	**n.d.† n (%)**						
Better	1 (1.1)	0	8 (8.8)	0	1 (1.1)	2 (2.2)	17 (18.7)	62 (68.1)
Weight	1 (1.1)	1 (1.1)	57 (62.6)	10 (11.0)	4 (4.4)	7 (7.7)	3 (3.3)	8 (8.8)
Condition	0	0	17 (18.7)	2 (2.2)	1 (1.1)	6 (6.6)	31 (34.1)	34 (37.4)
Energy	0	1 (1.1)	18 (19.8)	1 (1.1)	0	3 (3.3)	33 (36.3)	35 (38.5)
Pain	1 (1.1)	1 (1.1)	33 (36.3)	0	2 (2.2)	4 (4.4)	9 (9.9)	41 (45.1)
Bowel movements	0	1 (1.1)	58 (63.7)	4 (4.4)	2 (2.2)	1 (1.1)	12 (13.2)	13 (14.3)
Urinate	0	1 (1.1)	64 (70.3)	4 (4.6)	0	1 (1.1)	10 (11.0)	11 (12.1)
Shortness of breath	1 (1.1)	0	39 (42.9)	1 (1.1)	2 (2.3)	5 (5.5)	11 (12.1)	32 (35.2)
Walking	0	1 (1.1)	32 (35.2)	1 (1.1)	3 (3.3)	5 (5.5)	16 (17.6)	33 (36.3)
Moving	0	1 (1.1)	35 (38.5)	1 (1.1)	2 (2.2)	5 (5.5)	18 (19.8)	29 (31.9)
Appetite	0	2 (2.2)	55 (60.4)	1 (1.1)	2 (2.2)	6 (6.6)	9 (9.9)	16 (17.6)
Knowing what is	0	1 (1.1)	32 (35.2)	2 (2.2)	2 (2.2)	1 (1.1)	12 (13.2)	41 (45.1)

For peer review only

wrong								
Disease under control	0	1 (1.1)	10 (11.0)	0	0	2 (2.2)	15 (16.5)	63 (69.2)
Alive	0	1 (1.1)	2 (2.2)	2 (2.2)	1 (1.1)	1 (1.1)	13 (14.3)	71 (78.0)
Enjoy	0	2 (2.2)	20 (22.0)	0	0	2 (2.2)	13 (14.3)	54 (59.3)
Freedom	0	1 (1.1)	31 (34.1)	0	1 (1.1)	1 (1.1)	12 (13.2)	45 (49.5)
Cooking	0	2 (2.3)	51 (56.0)	1 (1.1)	4 (4.4)	2 (2.2)	14 (15.4)	17 (18.7)
Housework	0	1 (1.1)	51 (56.0)	2 (2.2)	5 (5.5)	5 (5.5)	10 (11.0)	17 (18.7)
Groceries	0	1 (1.1)	42 (46.2)	1 (1.1)	5 (5.5)	9 (9.9)	12 (13.2)	21 (23.1)
Wash and dress	0	2 (2.3)	51 (56.0)	0	0	2 (2.2)	14 (15.4)	22 (24.2)
Garden	0	1 (1.1)	48 (52.7)	2 (2.2)	1 (1.1)	8 (8.8)	10 (11.0)	21 (23.1)
Sports	0	1 (1.1)	46 (50.5)	7 (7.7)	2 (2.2)	9 (9.9)	7 (7.7)	19 (20.9)
Hobbies	0	1 (1.1)	39 (42.9)	1 (1.1)	2 (2.2)	4 (4.4)	13 (14.3)	31 (34.1)
Work	0	3 (3.3)	63 (69.2)	3 (3.3)	0	4 (4.4)	7 (7.7)	11 (12.1)
Driving	0	2 (2.2)	46 (50.2)	1 (1.1)	0	2 (2.2)	8 (8.8)	32 (35.2)
Outings	0	1 (1.1)	28 (30.8)	1 (1.1)	3 (3.3)	8 (8.8)	19 (20.9)	31 (34.1)
Visiting	0	3 (3.3)	28 (30.8)	1 (1.1)	4 (4.4)	6 (6.6)	19 (20.9)	30 (33.0)
Family life	0	4 (4.4)	39 (42.9)	0	0	2 (2.2)	12 (13.2)	34 (37.4)
Home	0	2 (2.3)	21 (23.1)	1 (1.1)	0	1 (1.1)	4 (4.4)	62 (68.1)
Independence	1 (1.1)	3 (3.3)	29 (31.9)	0	1 (1.1)	2 (2.2)	6 (6.6)	49 (53.8)

* Measurement failed: invalid answer due to two options filled in

† n.d. = not done. No answer was given.

As seen in Table 3, the number of missing values ranges from zero to four per item. The answer options with the lowest priorities were used the least, especially 'not at all important' and 'somewhat important'. Therefore, and also because on reflection the options 'somewhat' and 'moderately' were very close, we decided to remove the option 'moderately'.

Four dyads had a Spearman's rank-order correlation coefficient > 0.7: energy and condition (rs = .80); moving and walking (rs = .87); cooking and groceries (rs = .75); cooking and housekeeping (rs = .70) Therefore of these dyads, one item was removed (condition, moving and cooking), inspired by the information derived from the TSTI's.

Table 4 shows the items with mean impact scores, sorted per category and descending mean impact scores. From the categories with at least two items, the item with the lowest mean impact score was removed. To give participants still the opportunity to indicate their individual priorities, even when being a minority, we added an open option to add extra individual goals.

Table 4. Mean impact scores per category.

	University hospital			Regional teaching hospital		
**Goals**	**Applied**	**Importance**	**Mean**	**Applied**	**Importance**	**Mean**

For peer review only - http://bmjopen.bmj.com/site/about/guidelines.xhtml

	(%)	score (M)	impact score	(%)	score (M)	impact score
**Remain alive**						
Remain alive	98	3.70	3.62	75	2.64	1.90
**Controlling disease**						
Controlling disease	89	3.76	3.34	29	2.43	0.70
**Improving condition**						
Feeling better	91	3.71	3.38	71	2.73	1.94
Energy	80	3.40	2.72	50	2.23	1.12
Condition	*81*	*3.27*	*2.66*	*65*	*2.34*	*1.53*
Weight	*36*	*1.84*	*0.66*	*9*	*2.33*	*0.20*
**Alleviating complaints**						
Pain	63	3.59	2.26	44	2.72	1.20
Breath	57	3.39	1.92	38	2.64	0.99
Appetite	38	3.09	1.18	35	2.39	0.83
Bowel	35	2.88	1.03	29	2.47	0.71
Urinate	*29*	*2.92*	*0.83*	*17*	*2.67*	*0.46*
**Enjoying life**						
Enjoying life	78	3.75	2.91	31	2.53	0.78
**Improving/maintaining social functioning**						
Outing	69	3.23	2.23	27	2.11	0.57
Visiting	68	3.22	2.20	21	1.91	0.40
Family life	*55*	*3.67*	*2.03*	*5*	*2.80*	*0.13*
**Knowing what is wrong**						
Wrong	64	3.52	2.27	39	2.58	0.99
**Regaining/maintaining independence, freedom**						
Home	76	3.85	2.94	15	2.50	0.39
Independence	67	3.78	2.52	17	2.44	0.42
Freedom	*66*	*3.71*	*2.43*	*23*	*2.54*	*0.59*
**Improving daily functioning**						
Walking	64	3.33	2.14	54	2.57	1.38
Moving	*61*	*3.31*	*2.02*	*34*	*2.54*	*0.86*
Driving	48	3.63	1.75	14	2.13	0.31
Groceries	53	2.98	1.59	19	2.30	0.44
Wash/dress	43	3.53	1.52	26	2.52	0.65
Cooking	*43*	*3.11*	*1.33*	*15*	*1.75*	*0.27*
Housework	*43*	*2.90*	*1.26*	*20*	*1.86*	*0.38*
**Resuming work/hobbies**						
Hobbies	57	3.39	1.92	20	2.00	0.40
Garden	47	3.12	1.46	16	1.24	0.20
Sports	49	2.66	1.30	23	1.58	0.37
Work	*28*	*2.92*	*0.83*	*13*	*1.92*	*0.24*

The removed items are indicated in italic.

For peer review only

Repetition field test in regional teaching hospital

The field test was repeated in a regional teaching hospital with the same items, but with fewer answer options and the questions in two steps, as explained in the next paragraph. In the eight week inclusion period, 209 patients meeting the inclusion criteria were admitted on the wards. Of these patients, 56 were not approached for logistic reasons. 153 Were therefore approached for informed consent and 104 patients (67%) gave informed consent. The items with the lowest mean impact scores were the same for most categories, except for the categories independence/freedom, improving daily functioning and work/hobbies.

Splitting tool into two phases

Since some problems with understanding remained, especially the difficulties relating the goals to their own situation, as described in the TSTI, we decided to split the tool into two phases. In the first phase an inventory of subjects with problems or limitations was made. These could be problems/limitations at the moment of interview, at admission, or expected problems/limitations. In the second phase, only the importance was asked for the goals related to the subjects that applied. As this adaptation complicated the tool, we decided to use it as an interview-based tool. The item reduction and splitting into two phases, resulted in P-BAS HOP version 3.

5. TSTI with Version 3.

Version 3 was tested again with the TSTI in hospitalised older patients. The procedure was identical as in step 3, however as this version is only applicable as an interview version, this was done with an interviewer and observant. The observant only observed during the first step, and took over the interview role in the second and third steps.

Results

Eight participants participated in the TSTI about Version 3. Characteristics of the participants are displayed in the last column of Table 1.

General understanding

In general, the tool in two phases was well-understood. For example:

> Item *shortness of breath*, phase 1, step 1: *No, I have no problems with that, you know, shortness of breath.* (*A1*)
>
> Or:

For peer review only

> Item *shortness of breath*, phase 1, step 1: *Yes, that is present! And for that reason, I am admitted here. My oxygen was too low. And my carbon dioxide level is not good, much too high. Yes, complication of, yes.* (*A3*)

We shortened the instructions, but did not modify the content of the tool. This last adaptation led to the final questionnaire (Appendix 2). The completion of this baseline questionnaire took 5 to 24 minutes, with a median of 11 minutes.

DISCUSSION

The Patient Benefit Assessment Scale for Hospitalised Older patients (P-BAS HOP) was constructed as a tool that should be capable both to identify the goals and priorities of the individual older hospitalised patient and to measure the outcomes relevant to him regarding hospitalisation.

The items of the P-BAS HOP were based on interviews with hospitalised older patients. Including patients in the generation of patient reported outcomes is not self-evident and is even absent in many cases.[18] But even when patients are involved in the generation of outcomes, they still only reflect the priorities of the overall patient population and not the individual patient. Therefore, the major advantage of the P-BAS HOP is that patients can indicate their individual priorities, which also leads to individual benefit-scores.

Indicating individual priorities is also possible with the GAS, but the GAS is more time consuming, varying from 15-20 minutes for experienced assessors,[19] to 90 minutes per patient,[20] while the P-BAS HOP takes 5 to 24 minutes, with a median of 11 minutes. Moreover, for some older patients it might be difficult to formulate their own goals,[12] and the P-BAS HOP helps patients with examples of predefined goals.

More recently, models for goal based decision making were developed,[21-23] but these method are more suitable for clinical encounters to align treatment option with patient goals. The major advantage of the P-BAS HOP is that it is a more suitable and efficient tool to measure personalised outcomes in, for example, trials. It also could replace a diversity of existing tools, since it covers several dimension like symptoms, daily functioning, social functioning.

The pilot and field tests of the P-BAS HOP started already before we achieved complete saturation of goals in the qualitative interviews. Therefore, patients had the possibility to add goals during the TSTI. Several goals were added during the TSTI, which also appeared later in the qualitative interviews.[3] Still, the qualitative interviews revealed later some extra target complaints, which were not included in the P-BAS HOP, such as vomiting, dizziness and sweating. Yet, in the final version of the P-BAS HOP, patients still have the opportunity to add personal goals which were not mentioned before.

For peer review only

By using the mean impact score to reduce items, items considered least important by the overall sample were removed, though this does not take account of the priorities of individuals who deviate from the majority. For this reason the extra open option was added. Most removed items, based on mean impact score, were confirmed when repeated in the regional teaching hospital. The only exceptions were in the categories *improving daily functioning*, *resuming work/hobbies,* and *regaining/maintaining independence/freedom.*

In the categories improving daily functioning and resuming work/hobbies, the lowest priorities were 'housework' and 'work' in the first sample and 'driving' and 'gardening' in the second. Since driving and work were the second lowest priority in the second sample, the removal of housework and work could be justified.

In the category *regaining/maintaining independence/freedom* priorities in both hospitals were entirely opposite. We therefore have to conclude that we were too early to remove the item freedom. It is unclear whether these differences are caused by different contexts or because the field test in the regional hospital was after splitting the questionnaire into two phases and therefore the questions were altered.

Limitations

The P-BAS HOP is only tested in hospitalised patients without cognitive impairment. It is therefore unknown if it is suitable in other contexts and it might be too complex for patients with cognitive impairment. In addition, the P-BAS HOP is only tested in the Netherlands and the translated English version has not yet been tested. Therefore, it is unknown whether the P-BAS HOP is applicable in other languages and cultures.

The TSTI gave valuable insights into the understanding of the questionnaire and the completion behaviour of the participants. Many adaptations were made, but it proved difficult to make the questionnaire understandable for all patients. These kinds of difficulties were seen in various examples where the TSTI was used.[16, 24-26] Unfortunately, the final version is only suitable to be completed with an interviewer and not as a self-administered questionnaire. The TSTI gave a first indication of the content validity, but further quantitative research into the construct validity, reliability and responsivity is needed.

Conclusions

The Patient Benefit Assessment Scale for Hospitalised Older Patients (P-BAS HOP) is a potentially suitable interview-based tool to identify the priorities and relevant outcomes of the individual older hospitalised adult. Based on these data it is possible to compute an individual Patient Benefit Index, which is an overall value between 0 (no benefit) and 3 (maximal benefit), which reflects the

For peer review only

achievement of the goals weighted by the importance. Further quantitative research is needed to investigate the construct validity, reliability and responsiveness.

Acknowledgements We would like to thank all patients who participated in the interviews for sharing their stories with us and their feedback on the different versions of the P-BAS HOP. We thank the panel members for their feedback on the draft version. We would also like to thank all the research assistants and students for the many interviews they conducted, especially Gerdine Boot for all the patients she interviewed in the regional teaching hospital. We would like to thank Daniël Bosold, Janet Vroomen, Caroline Mutsaers, Margaretha Schenkeveld and Anna Pot for their role in the translation process of the P-BAS HOP.

Funding This study was funded by an unrestricted grant from the Dutch Foundation for Effective Elderly Care and the University of Groningen.

Author's contribution MJvdK designed the study. MJvdK conducted the Three Step Test Interviews (TSTI's). MJvdK coded the data of the TSTI's. MJvdK and SEdR regularly discussed the codes and the progress and adaptations of the different versions of the P-BAS HOP. SEdR supervised the field test in the University hospital, BCvM in the Regional teaching hospital. MJvdK wrote the first draft of the manuscript, GJD, BCvM and SEdR contributed significantly to subsequent manuscript revisions. All authors have read and approved the final version of the manuscript.

Competing interests None declared

Data sharing All data relevant to the study are included in the article

Patient consent All patients gave informed consent

Ethical approval

The Medical Ethics Research Committee of the UMCG (file number M16.192615 and M16.199647) confirmed that the Medical Research Involving Human Subjects Act did not apply to the research project. Official approval by the committee was therefore not required.

For peer review only

REFERENCES

1 Reuben DB, Tinetti ME. Goal-oriented patient care--an alternative health outcomes paradigm. *N Engl J Med* 2012;366:777-9 doi:10.1056/NEJMp1113631.

2 Tinetti ME, Naik AD, Dodson JA. Moving from disease-centered to patient goals-directed care for patients with multiple chronic conditions: Patient value-based care. *JAMA Cardiology* 2016;1:9-10 doi:10.1001/jamacardio.2015.0248.

3 Van der Kluit MJ, Dijkstra GJ, de Rooij S,E. Goals of older hospitalised patients: a qualitative descriptive study. *BMJ open* 2019;9:e029993- doi:10.1136/bmjopen-2019-029993.

4 Vermunt NP, Harmsen M, Elwyn G, et al. A three-goal model for patients with multimorbidity: A qualitative approach. *Health Expect* 2018;21:528-38 doi:10.1111/hex.12647 [doi].

5 Donnelly C, Carswell A. Individualized outcome measures: a review of the literature. *Can J Occup Ther* 2002;69:84-94.

6 Mangin D, Stephen G, Bismah V, et al. Making patient values visible in healthcare: a systematic review of tools to assess patient treatment priorities and preferences in the context of multimorbidity. *BMJ Open* 2016;6:e010903- doi:10.1136/bmjopen-2015-010903.

7 van Seben R, Reichardt L, Smorenburg S, et al. Goal-Setting Instruments in Geriatric Rehabilitation: A Systematic Review. *J Frailty Aging* 2017;6:37-45 doi:10.14283/jfa.2016.103.

8 Fried TR, Tinetti M, Agostini J, et al. Health outcome prioritization to elicit preferences of older persons with multiple health conditions. *Patient Educ Couns* 2011;83:278-82 doi:10.1016/j.pec.2010.04.032.

9 Stolee P, Awad M, Byrne K, et al. A multi-site study of the feasibility and clinical utility of Goal Attainment Scaling in geriatric day hospitals. *Disabil Rehabil* 2012;34:1716-26 doi:10.3109/09638288.2012.660600 [doi].

10 Battle CC, Imber SD, Hoehn-Saric R, et al. Target complaints as criteria of improvement. *Am J Psychother* 1966;20:184-92 doi:10.1176/appi.psychotherapy.1966.20.1.184 [doi].

11 Hesbacher PT, Rickels K, Weise C. Target symptoms: a promising improvement criterion in psychiatric drug research. *Arch Gen Psychiatry* 1968;18:595-600.

12 Schulman-Green DJ, Naik AD, Bradley EH, et al. Goal setting as a shared decision making strategy among clinicians and their older patients. *Patient Educ Couns* 2006;63:145-51 doi:S0738-3991(05)00275-2 [pii].

13 Augustin M, Reich C, Schaefer I, et al. Development and validation of a new instrument for the assessment of patient-defined benefit in the treatment of acne. *J Dtsch Dermatol Ges* 2008;6:113-20 doi:10.1111/j.1610-0387.2007.06540.x.

14 De Vet HCW, Terwee CB, Mokkink LB, et al. Measurement in Medicine. A Practical Guide. Cambridge: Cambridge University Press 2011.

15 Beaton DE, Bombardier C, Guillemin F, et al. Guidelines for the Process of Cross-Cultural Adaptation of Self-Report Measures. *Spine* 2000;25:3186-91 doi:10.1097/00007632-200012150-00014.

16 Pool JJM, Hiralal S, Ostelo, Raymond W J G, et al. Added value of qualitative studies in the development of health related patient reported outcomes such as the Pain Coping and Cognition List in patients with sub-acute neck pain. *Man Ther* 2010;15:43-7 doi:10.1016/j.math.2009.05.010.

17 Van Der Veer K, Ommundsen R, Hak T, et al. Meaning Shift of Items in Different Language Versions. A Cross-National Validation Study of the Illegal Aliens Scale. *Quality and Quantity* 2003;37:193-206.

For peer review only

18 Wiering B, de Boer D, Delnoij D. Patient involvement in the development of patient-reported outcome measures: a scoping review. *Health Expect* 2017;20:11-23 doi:10.1111/hex.12442 [doi].

19 Stolee P, Rockwood K, Fox RA, et al. The use of goal attainment scaling in a geriatric care setting. *J Am Geriatr Soc* 1992;40:574-8.

20 Toto PE, Skidmore ER, Terhorst L, et al. Goal Attainment Scaling (GAS) in geriatric primary care: a feasibility study. *Arch Gerontol Geriatr* 2015;60:16-21 doi:10.1016/j.archger.2014.10.022 [doi].

21 Elwyn G, Vermunt NPCA. Goal-Based Shared Decision-Making: Developing an Integrated Model. *Journal of Patient Experience* 2019:1-9 doi:10.1177/2374373519878604.

22 Naik AD, Dindo LN, Van Liew JR, et al. Development of a Clinically Feasible Process for Identifying Individual Health Priorities. *J Am Geriatr Soc* 2018;66:1872-9 doi:10.1111/jgs.15437 [doi].

23 Tinetti ME, Naik AD, Dindo L, et al. Association of Patient Priorities-Aligned Decision-Making With Patient Outcomes and Ambulatory Health Care Burden Among Older Adults With Multiple Chronic Conditions: A Nonrandomized Clinical Trial. *JAMA Intern Med* 2019;179:1688-97 doi:10.1001/jamainternmed.2019.4235 [doi].

24 Jansen H, Hak T. The Productivity of the Three-step Test-interview (TSTI) Compared to an Expert Review of a Self-administered Questionnaire on Alcohol Consumption. *Journal of official statistics* 2005;21:103-20.

25 Liu RD, Buffart LM, Kersten MJ, et al. Psychometric properties of two physical activity questionnaires, the AQuAA and the PASE, in cancer patients. *BMC Med Res Methodol* 2011;11:30,2288-11-30 doi:10.1186/1471-2288-11-30 [doi].

26 van Alphen A, Westerman MJ, Visschedijk JH, et al. A qualitative study of Falls Efficacy Scale-International/Hips. What do we measure?. *Tijdschr Gerontol Geriatr* 2013;44:3-11 doi:10.1007/s12439-013-0005-9 [doi].

For peer review only

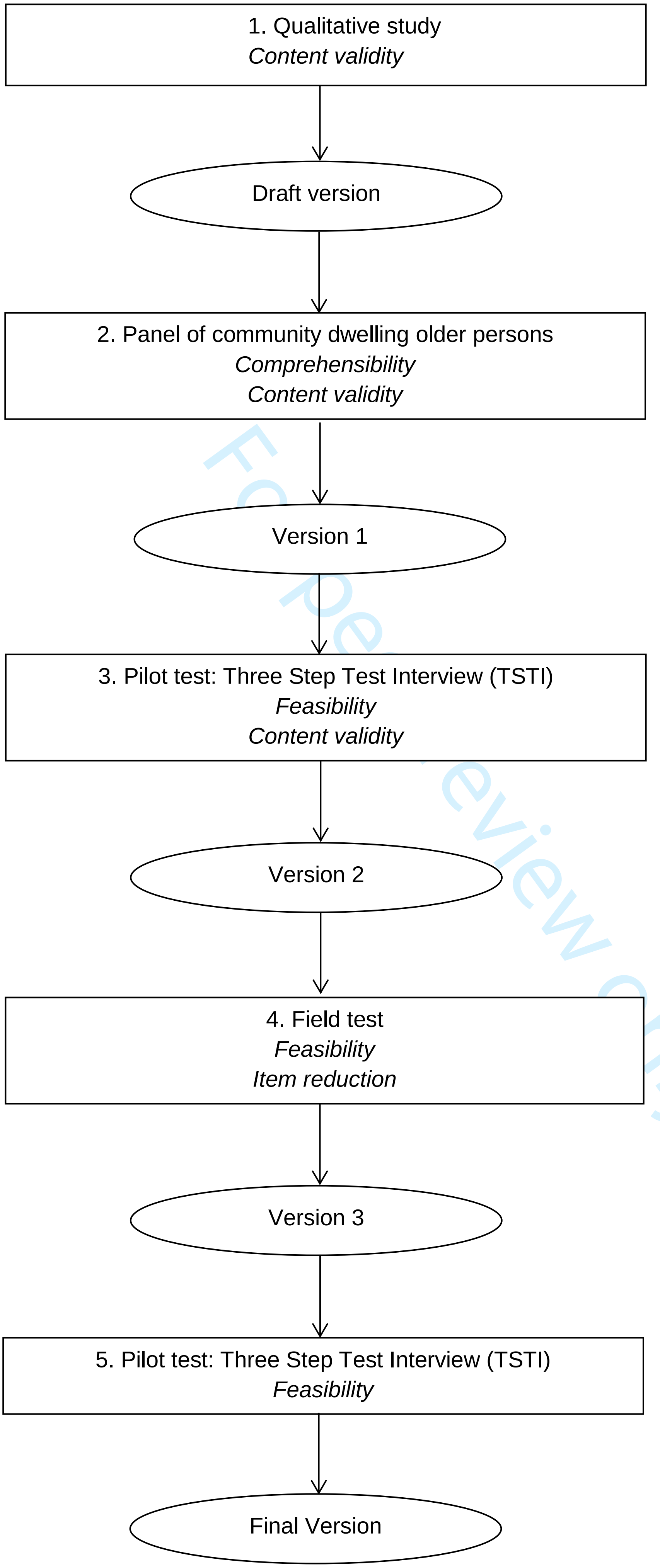

For peer review only - http://bmjopen.bmj.com/site/about/guidelines.xhtml

Appendix 1. Version 1. Patient Benefit Assessment Scale

Hospitalisation goals

The following questions cover how important the goals below are for you during your current hospitalisation.

Can you indicate how important each goal below is for you? You can choose from: 'not at all', 'somewhat', 'moderately' 'quite', or 'very'. If a goal doesn't apply to you, for example because you don't have difficulty with the listed problem (for example with bowel movements, or shortness of breath) or because you don't have a garden, then choose 'does not apply to me'.

	How important is it for you that through this hospitalisation…	Not at all	Somewhat	Moderately	Quite	Very	Does not apply to me
1	You feel better						
2	You regain weight						
3	Your condition improves						
4	You have more energy						
5	You can walk better						
6	You move easier						
7	You can do housework						
8	You can cook						
9	You can do the groceries						
10	You can garden						
11	You can take pleasure in eating						
12	You can wash and dress yourself						
13	You can exercise or participate in sports						
14	You have no pain						
15	You have normal bowel movements						
16	You have less shortness of breath						
17	Your disease is under control						
18	You remain alive						
19	You can enjoy life						
20	You regain your freedom						
21	You can resume your (volunteer) work						
22	You can go on outings						
23	You can visit friends or family						
24	You can take a short break						
25	You know the cause of your complaints						
26	You can go back home						
27	You regain your independence						

For peer review only

Evaluation of hospitalisation goals

In the beginning of your hospitalisation you indicated how important various goals for you were. Can you indicate for each of the goals below how much the hospitalisation has helped to achieving the goal? You can indicate whether the hospitalisation helped you 'not at all', 'somewhat', 'moderately' 'quite', or 'very'. If a goal didn't apply to you, for example because you didn't have difficulty with the listed problem (for example with bowel movements, or shortness of breath) or because you have no garden, then choose 'did not apply to me'.

	The hospitalisation helped me to...	Not at all	Somewhat	Moderately	Quite	Very	Did not apply to me
1	Feel better						
2	Regain weight						
3	Improve my condition						
4	Have more energy						
5	Walk better						
6	Move easier						
7	Do the housework						
8	Cook						
9	Do the groceries						
10	Garden						
11	Take pleasure in eating						
12	Wash and dress myself						
13	Exercise or Participate in sports						
14	Have no pain						
15	Have normal bowel movements						
16	Have less shortness of breath						
17	Keep my disease under control						
18	Remain alive						
19	Enjoy life						
20	Regain my freedom						
21	Resume my volunteer work						
22	Go on outings						
23	Visit family or friends						
24	Can take a short break						
25	Know what the cause of my complaints is/was						
26	Go back to my home						
27	Regain my independence						

For peer review only

Appendix 2. Final Version Patient Benefit Assessment Scale
Hospitalisation Goals

People differ in what they wish to achieve with a hospitalisation. They have different goals. This depends on what they suffer from, what they are hospitalised for, and what they find important in life.

I am now going to mention some subjects that may be important to you during this hospitalisation. Can you say whether each applies to you?

A subject applies to you if you experience or anticipate problems or limitations and this applies to your life. For people who, for example, are short of breath, the subject shortness of breath probably applies, but not for others. For others they may be struggling to enjoy life because of their illness, but if you experience no problems with it, then this doesn't apply to you.

Instruction for the interviewer: Circle the number for the subject that applies to the participant.

If a participant asks what you mean by a subject, say:
Are you experiencing problems with [subject] now, or when you were admitted, or are you expecting problems with it because of your illness or hospitalisation?
For the functional subjects, prefer not to use the word 'problem', but rather 'trouble' or 'limitation'.

Now follow the subjects, you should indicate whether each subject applies to you or not:
1. Wanting to feel better
Explanation if necessary: do you feel sick or miserable and would you like to feel better?
2. Energy
3. Pain
4. Bowel movements
5. Shortness of breath
6. Walking
7. Appetite
8. Unclarity about what is wrong with me
Explanation if necessary: you may be wondering what is wrong with you. If this was already clear for you when you were admitted then this does not apply.
9. Controlling my disease
Explanation if necessary: Do you suffer from a disease that needs to be controlled?
10. Remain alive
Explanation if necessary: Was your life in danger when you were admitted or do you think that this hospitalisation must contribute to remain alive?
11. Enjoying life
12. Housework
13. Groceries
14. Washing and dressing myself
15. Gardening
16. Exercise or Sports
17. Hobbies
18. Driving
19. Going on outings
20. Visiting family or friends

For peer review only

21. Return back to my home
Explanation if necessary: Are you unsure whether you can return to your own home?
22. Independence
23. Are there other themes which are important to you during this hospitalisation that I have not mentioned yet? If so, which?

Now that we have identified the themes that apply to you, I am going to ask to what extent they are goals during this hospitalisation.

Can you indicate for each goal that I mention how important it is during this hospitalisation? You can choose from 'not at all important', 'moderately important' 'quite important', or 'very important'.

For some goals you may still say that they do not apply, for example because they do not matter during this hospitalisation. Then you indicate "does not apply to me now".

Instruction to interviewer: Read only the goals which you have circled.

For peer review only

		does **not apply** to me now	**not at all** important	**somewhat** important	**quite** important	**very** important
1	How important is it for you that you **feel better again** as a result of this hospitalisation	☐	☐	☐	☐	☐
2	How important is it for you that you **have more energy** as a result of this hospitalisation	☐	☐	☐	☐	☐
3	How important is it for you that you **have less pain** as a result of this hospitalisation	☐	☐	☐	☐	☐
4	How important is it for you that you **have normal bowel movements again** as a result of this hospitalisation	☐	☐	☐	☐	☐
5	How important is it for you that you **are less short of breath** as a result of this hospitalisation	☐	☐	☐	☐	☐
6	How important is it for you that you can **walk better again** as a result of this hospitalisation	☐	☐	☐	☐	☐
7	How important is it for you that you **regain your appetite** as a result of this hospitalisation	☐	☐	☐	☐	☐
8	How important is it for you that you **know what is wrong with you** as a result of this hospitalisation	☐	☐	☐	☐	☐
9	How important is it for you that **your disease is under control** as a result of this hospitalisation	☐	☐	☐	☐	☐
10	How important is it for you that you **remain alive** as a result of this hospitalisation	☐	☐	☐	☐	☐
11	How important is it for you that you can **enjoy life again** as a result of this hospitalisation	☐	☐	☐	☐	☐
12	How important is it for you that you can **do housework again** as a result of this hospitalisation	☐	☐	☐	☐	☐

		does **not apply** to me now	**not at all** important	**somewhat** important	**quite** important	**very** important
13	How important is it for you that you can **do the groceries again** as a result of this hospitalisation	☐	☐	☐	☐	☐
14	How important is it for you that you **can wash and dress yourself again** as a result of this hospitalisation	☐	☐	☐	☐	☐
15	How important is it for you that you **can garden again** as a result of this hospitalisation	☐	☐	☐	☐	☐
16	How important is it for you that you **can exercise or participate in sports again** as a result of this hospitalisation	☐	☐	☐	☐	☐
17	How important is it for you that you **can exercise your hobbies again** as a result of this hospitalisation	☐	☐	☐	☐	☐
18	How important is it for you that you **can drive again** as a result of this hospitalisation	☐	☐	☐	☐	☐
19	How important is it for you that you **go on outings again** as a result of this hospitalisation	☐	☐	☐	☐	☐
20	How important is it for you that you **can visit family or friends again** as a result of this hospitalisation	☐	☐	☐	☐	☐
21	How important is it for you that you **can return to your own home again** as a result of this hospitalisation	☐	☐	☐	☐	☐
22	How important is it for you that you **regain your independence** as a result of this hospitalisation	☐	☐	☐	☐	☐
23	How important is it for you that you ___________________ as a result of this hospitalisation	☐	☐	☐	☐	☐
24	Do you have any goals I haven't mentioned yet? If so: How important is it to you that___________________________ as a result of this hospitalisation	☐	☐	☐	☐	☐

Evaluation of hospitalisation goals

In the beginning of your hospitalisation you indicated how important various goals for you were. A goal is something you want to achieve with a hospitalisation. Some goals you may have achieved, others maybe not or not entirely.

Can you indicate for each of the goals below how much the hospitalisation has helped to achieving the goal? You can indicate whether the hospitalisation has helped you 'not at all', 'moderately', 'quite', or 'completely'.

Only the goals which applied at baseline are evaluated with the participant.

		Not at all	Somewhat	Quite	Completely
1	Because of the hospitalisation I **feel better again**	☐	☐	☐	☐
2	Because of the hospitalisation I have **more energy**	☐	☐	☐	☐
3	Because of the hospitalisation I have **no more pain**	☐	☐	☐	☐
4	Because of the hospitalisation I have **normal bowel movements again**	☐	☐	☐	☐
5	Because of the hospitalisation I am **less short of breath**	☐	☐	☐	☐
6	Because of the hospitalisation I **walk better again**	☐	☐	☐	☐
7	Because of the hospitalisation I **regained appetite**	☐	☐	☐	☐
8	Because of the hospitalisation I **know what is/ was wrong with me**	☐	☐	☐	☐
9	Because of the hospitalisation **my disease is under control**	☐	☐	☐	☐
10	Because of the hospitalisation I **remained alive**	☐	☐	☐	☐
11	Because of the hospitalisation I **enjoy life again**	☐	☐	☐	☐
12	Because of the hospitalisation I **do my housework again**	☐	☐	☐	☐
13	Because of the hospitalisation I **do the groceries again**	☐	☐	☐	☐
14	Because of the hospitalisation I **wash and dress myself again**	☐	☐	☐	☐
15	Because of the hospitalisation I **garden again**	☐	☐	☐	☐
16	Because of the hospitalisation I **participate in sports again**	☐	☐	☐	☐

For peer review only

		Not at all	Somewhat	Quite	Completely
17	Because of the hospitalisation I **can resumed my hobbies**	☐	☐	☐	☐
18	Because of the hospitalisation I **drive again**	☐	☐	☐	☐
19	Because of the hospitalisation I **go on outings again**	☐	☐	☐	☐
20	Because of the hospitalisation I **visit family or friends again**	☐	☐	☐	☐
21	Because of the hospitalisation I **am back in my own home**	☐	☐	☐	☐
22	Because of the hospitalisation I **regained independence**	☐	☐	☐	☐
23	Because of the hospitalisation ___________________________	☐	☐	☐	☐

For peer review only

BMJ Open

Development of a new tool for the assessment of patient defined benefit in hospitalised older patients: the Patient Benefit Assessment Scale for Hospitalised Older Patients (P-BAS HOP)

Journal:	*BMJ Open*
Manuscript ID	bmjopen-2020-038203.R2
Article Type:	Original research
Date Submitted by the Author:	01-Oct-2020

Complete List of Authors:	van der Kluit, Maria; University Medical Centre Groningen, University Center for Geriatric Medicine<br>Dijkstra, Geke; Department of Health Sciences, Applied Health Research<br>van Munster, Barbara; University of Groningen, University Medical Center Groningen, University Center for Geriatric Medicine; Gelre Hospitals, Geriatrics<br>De Rooij, Sophia; University Medical Center Groningen, University Center for Geriatric Medicine; Medical Spectrum Twente, Medical School Twente

**Primary Subject Heading**:	Geriatric medicine
Secondary Subject Heading:	Communication, Health services research, Research methods
Keywords:	GERIATRIC MEDICINE, QUALITATIVE RESEARCH, Quality in health care < HEALTH SERVICES ADMINISTRATION & MANAGEMENT

SCHOLARONE™
Manuscripts

I, the Submitting Author has the right to grant and does grant on behalf of all authors of the Work (as defined in the below author licence), an exclusive licence and/or a non-exclusive licence for contributions from authors who are: i) UK Crown employees; ii) where BMJ has agreed a CC-BY licence shall apply, and/or iii) in accordance with the terms applicable for US Federal Government officers or employees acting as part of their official duties; on a worldwide, perpetual, irrevocable, royalty-free basis to BMJ Publishing Group Ltd ("BMJ") its licensees and where the relevant Journal is co-owned by BMJ to the co-owners of the Journal, to publish the Work in this journal and any other BMJ products and to exploit all rights, as set out in our licence.

The Submitting Author accepts and understands that any supply made under these terms is made by BMJ to the Submitting Author unless you are acting as an employee on behalf of your employer or a postgraduate student of an affiliated institution which is paying any applicable article publishing charge ("APC") for Open Access articles. Where the Submitting Author wishes to make the Work available on an Open Access basis (and intends to pay the relevant APC), the terms of reuse of such Open Access shall be governed by a Creative Commons licence – details of these licences and which Creative Commons licence will apply to this Work are set out in our licence referred to above.

Other than as permitted in any relevant BMJ Author's Self Archiving Policies, I confirm this Work has not been accepted for publication elsewhere, is not being considered for publication elsewhere and does not duplicate material already published. I confirm all authors consent to publication of this Work and authorise the granting of this licence.

For peer review only

Development of a new tool for the assessment of patient defined benefit in hospitalised older patients: the Patient Benefit Assessment Scale for Hospitalised Older Patients (P-BAS HOP)

Authors:

1. Maria Johanna van der Kluit, MSc RN (Corresponding author)

   University of Groningen, University Medical Center Groningen, University Center for Geriatric Medicine, Hanzeplein 1, 9700 RB Groningen, The Netherlands

   m.j.van.der.kluit@umcg.nl

   +31503613921

2. Geke J. Dijkstra, PhD

   University of Groningen, University Medical Center Groningen, Department of Health Sciences, Applied Health Research, Groningen, The Netherlands

   g.j.dijkstra@umcg.nl

3. Barbara C. van Munster, MD PhD

   University of Groningen, University Medical Center Groningen, University Center for Geriatric Medicine, Groningen, The Netherlands.

   Department of Geriatrics, Gelre Hospitals, Apeldoorn, The Netherlands.

   b.c.van.munster@umcg.nl

4. Sophia E. de Rooij, MD PhD

   University of Groningen, University Medical Center Groningen, University Center for Geriatric Medicine, Groningen, The Netherlands

   Medical Spectrum Twente, Medical School Twente, Enschede, The Netherlands

   sejaderooij@gmail.com

Words: 4784

Words abstract: 299

References: 31

Figures: 1

Tables: 4

For peer review only

ABSTRACT

Objectives: To support the shift from disease-oriented towards goal-oriented care, we aimed to develop a tool which is capable both to identify priorities of an individual older hospitalised patient and to measure the outcomes relevant to him.

Design: Mixed methods design with open interviews, Three Step Test Interviews (TSTI), and a quantitative field test.

Setting: University teaching hospital and a regional teaching hospital.

Participants: Hospitalised patients ages 70 years and older.

Results: The Patient Benefit Assessment Scale for Hospitalised Older Patients (P-BAS HOP) consists of a baseline questionnaire and an evaluation questionnaire. Items were based on 15 qualitative interviews with hospitalised older patients. Feedback from a panel of four community-dwelling older persons resulted in some adaptations to wording and one additional item. Twenty-six hospitalised older patients participated in TSTIs with Version 1 of the baseline questionnaire, revealing indications for a good content validity and barriers in completion behaviour, global understanding, and understanding of individual items, which were solved with several adaptations. Four additions were made by participants. After TSTIs with ten patients with the evaluation questionnaire, one adaptation was made. A field test with 91 hospitalised older patients revealed a small number of missing values.

To enhance the feasibility, the number of items was reduced from 32 to 23, based on correlations and mean impact score. The field test was repeated with 104 other patients in a regional teaching hospital. To enhance the understanding, the tool was split into two phases. This version was tested with TSTIs with eight patients and appeared to be understandable. The final version was an interview-based tool and took about 11 minutes to complete.

Conclusions: The P-BAS HOP is a potentially suitable tool to identify priorities and relevant outcomes of the individual patient. Further research is needed to investigate its validity, reliability and responsiveness.

Keywords Geriatric medicine; Older adults; Hospitalisation; Patient perspective; Goal setting; Patient-reported outcomes; Tool; Value-Based Health Care

ARTICLE SUMMARY

Strengths and limitations of this study

- The content of the P-BAS HOP is based on open interviews with hospitalised older patients.
- Patients are able to indicate their individual outcome priorities.

For peer review only

- The P-BAS HOP is tested intensively in the target population with Three Step Test Interviews. This gave valuable insights into the understanding of the tool and the completion behaviour of the participants.
- The current version of the P-BAS HOP is only suitable to be completed with an interviewer and not as a self-administered questionnaire.
- It is unknown whether the P-BAS HOP is feasible in other healthcare systems, languages and cultures than in the Netherlands.

For peer review only

BACKGROUND

To fit the needs of the ageing population, and patients with multiple chronic diseases, a shift is recommended from disease-oriented towards goal-oriented care. Older patients with multimorbidity may be more interested in more personal goals such as for them important symptoms, functional status and social functioning than in traditional outcomes such as survival and biomarkers,[1, 2] but these goals and outcomes differ per individual.[3, 4] When care would be systematically evaluated by personal goal-oriented outcomes, a tool is needed which is capable both to identify the priorities of the individual patient and to measure the outcomes relevant to him.

Three literature reviews[5-7] into tools used to assess patient outcome priorities in the context of multimorbidity revealed a few potentially useful tools. Tools only suitable for specific activities, such as the Canadian Occupational Performance Measure (COPM),[5, 7] Self-Identified Goals Assessment (SIGA),[7] Assessment of Motor and Process Skills (AMPS) and McMaster Toronto Arthritis (MACTAR)[5] were ignored. A general tool is the Outcome Prioritization Instrument,[6, 8] which is suitable to elicit four patient priorities, but these priorities are still very global and it remains unclear how to evaluate them after treatment. Another tool is the Goal Attainment Scaling (GAS)[5, 7] which is designed to set and evaluate individualized goals and outcomes. Disadvantages of the GAS are that it can be too challenging for patients to articulate their own goals and that it is time consuming.[9] The International Classification of Functioning, Disability and Health (ICF) framework for goal setting is used to categorize patient goals set in semi-structured interviews, but has still the same disadvantages as the GAS and has, in addition, a very poor responsiveness.[7] Finally, with the Target Complaints,[5] the patient defines target complaints as those problems for which help was sought. These complaints are scored at the beginning and at the end of the treatment by the patient, or the patient rates the degree of improvement.[10, 11] The Target Complaints is individualized and patient-centred. However, it focuses solely on problems and not on goals. Disadvantages for the GAS, ICF and Target Complaints could be that for some older patients it might be difficult to formulate their own goals and problems because many older persons are not accustomed to defining and discussing personal goals and prompting is often necessary.[12] The quality of the answers is therefore dependent on the interviewer's experiences and techniques.

For this reason, another method of defining patient-defined goals and outcomes was sought and found in the literature about treatment of acne. Augustin et al.[13] developed a tool consisting of two parts: 1) a baseline questionnaire to assess the importance of various predefined goals, based on themes derived from qualitative interviews in patients with acne and 2) an evaluation questionnaire to evaluate the extent to which treatment helped to achieve these goals. Based on these data it is possible to compute an individual Patient Benefit Index. This is an overall value between 0 (no benefit) and 4 (maximal benefit), which reflects the achievement of the goals weighted by the

For peer review only

importance.[13] The advantage of this tool is the insight into the individualized patient perspective, together with standardisation.

The aim of this study was to develop a tool to inventory individual goals and benefits of older hospitalised patients, based on the model of Augustin et al.[13] This article presents its development, early testing and adaptations.

METHODS

The steps used to develop the Patient Benefit Assessment Scale for Hospitalised Older Patients (P-BAS HOP) are based on the steps of De Vet et al.[14] and outlined in Figure 1. After each step, the tool was adapted. The steps are explained in the following sections. For the readability, the methods and results of each step are alternated. The P-BAS was developed and tested in Dutch. The P-BAS was translated into English in a translation - back translation procedure involving four translators (two native English, two native Dutch), a language professional and authors MJvdK and GD.[15]

Patient and public involvement

Patients and public were involved in the generation of the items, the importance and relevance of the items and the assessment of the feasibility and understanding of the tool.

Figure 1. Development of the P-BAS HOP

1. Qualitative study

Firstly, open interviews with hospitalised older medical and surgical patients about their goals regarding their hospitalisation were performed. The description of these goals is published elsewhere.[3] These goals were then coded inductively and transformed into questionnaire items, and the first draft of the P-BAS HOP was then constructed, consisting of a baseline questionnaire and an evaluation questionnaire.

2. Panel of community-dwelling older persons

The first draft version was proposed by e-mail to a panel of community-dwelling older persons to assess the comprehensibility and relevance of the items and the tool and ask for omissions or redundancies.

Results

Four community-dwelling older persons gave written feedback on the draft version of the tool. This led to adaptation in wording of the introduction text and to some items which were not clear enough

For peer review only

or could be interpreted in multiple ways. An example of an item that was adapted: for the item 'to be able to eat', it was not clear if it concerned the instrumental activity of eating or concerned appetite. Therefore, the item was changed into 'can take pleasure in eating'.

Further, one additional item was added, namely 'to wash and dress yourself' and the sequence of two items was changed. Version 1 of the P-BAS HOP is shown in Appendix 1.

3. Pilot test: Three Step Test Interview (TSTI).

The adapted tool (Version 1, Appendix 1) was tested with the TSTI[16, 17] in older hospitalised patients. The TSTI is a type of cognitive interview suitable to assess how people interpret a questionnaire, its different items and what kind of strategies they use in responding to them. The TSTI consists of the following steps:

Step 1: Concurrent thinking aloud

The participant completed version 1 of the P-BAS HOP while thinking aloud. The interviewer observed, made notes of the participant's behaviour (hesitations, skipping questions, corrections) and verbalized thoughts. However, the interviewer did not talk, or intervene. The instructions for the participant were: Please fill in this questionnaire and try to think aloud about what your thoughts are while reading the questions and choosing the right response category.

Step 2: Retrospective interview

With the retrospective interview any gaps from the first step were filled in. Every behaviour and thought from the observation of which the interviewer wanted further information, was clarified.

Step 3: Semi-structured interview

An in-depth interview was conducted, aimed at eliciting the participant's considerations and opinions. The participant was given the opportunity to explain behaviour, actions or thoughts that he had in the previous steps. The participant was asked how he understood different items, was asked for any omissions and his opinion about filling in the questionnaire. The participant was also asked to explain his goals in his own words in order to perform a first content validation of the P-BAS HOP.

Participants

Eligible participants of the TSTI were 70 years and older; planned or unplanned hospitalised on medical or surgical wards of a university teaching hospital in the Netherlands; able to speak and understand Dutch and were without cognitive impairment. Inclusion criteria were verified with the staff nurse, and patients were then approached by the interviewer (MJvdK). Participants were completely anonymous, no list with names or other identifying data was made, nor did the researchers have access to medical records. Participants gave verbal consent to the interview and audio recording.

For peer review only

Data analysis

Data gathering and data analysis were alternated. Interviews were audio recorded and transcribed verbatim. All remarks were then organised by question and step. After that, the data were coded by MJvdK and grouped into categories. The tool was adapted several times after the feedback until it was considered feasible and understandable.

The TSTI was repeated with the evaluation questionnaire. This was done at patient discharge.

Results

Sample baseline questionnaire

Twenty-six older hospitalised patients participated in the TSTI. Characteristics of the participants are displayed in the second column of Table 1.

Table 1. Participants Three Steps Test Interview (TSTI) baseline questionnaire version 1, evaluation questionnaire version 1 and baseline questionnaire version 3.

	Baseline questionnaire version 1 (n=26)	Evaluation questionnaire version 1 (n=10)	Baseline questionnaire version 3 (n=8)
Characteristic	n	n	n
Gender			
Male	19	8	4
Female	7	2	4
Age (years)			
70-79	18	8	7
80-89	7	2	0
90-99	1	0	1
Native language			
Dutch	14	7	7
Local dialect	10	2	1
Frisian	2	0	0
Foreign language	0	1	0
Educational level*			
Low	8	5	2

For peer review only

Middle	10	4	5
High	8	1	1
Admission reason†			
Cardiac problems	9	1	1
Pulmonary problems	7	3	2
Bowel problems	2	2	1
Fever/ infection	2	2	0
Vascular surgery	2	1	0
Cancer	2	0	0
Accident/fracture(s)	2	0	1
Kidney problems	0	1	1
Syncope	0	0	1
Ulcera	0	0	1

* Definition educational level: Low= no education, primary school, basic vocational training; Middle = secondary education, vocational training; High = bachelor, master

† Reason according to the patient

Coding categories

The codes were sorted into the following categories: Completion behaviour, Global understanding, Understanding and reactions regarding individual items, Additions, General evaluation, and Content validity.

Completion behaviour

With 'completion behaviour' is meant the behaviour participants showed when completing the questionnaire. Observations revealed that many participants skipped the instruction text partly or even completely. For some participants, reading these instructions was demanding, others did not understand how a table works and where to place a mark. Adding an example table with instructions showing how and where to place a mark brought no improvements. In the final version boxes to tick were included in the table.

Global understanding

Many examples of correct understanding were coded. For example:

> Item *regaining weight*, step 1: *Oops, dear, pooh, let's see, that is certainly important, because I have lost weight lately since I had not been feeling well for a while, that is, not*

For peer review only

shortness of breath or anything, but, very tired, listless. So, a little weight gain is important. (P9)

Or:

Item *walking*, step 1: *Well, I walk well. Doesn't apply to me.* (*P17*)

A few participants interpreted the questions as if it was an evaluation of their current level of functioning. For example:

Item *energy*, step 2: *I: You have filled in 'not at all' in 'you have more energy'. What is the reason that you just ...? P: Because I feel lethargic. That is what I mean to say. I used to be a very energetic person. (…) That is gone. (…) That is what I mean by that question. I: Yes, so you actually filled in how you are feeling now. P: Yes, now. At the time. I: Yes, so you say .. P: Not from last year or half a year ago. They are snapshots, aren't they? That was what you meant, right? (...) I: And when I ask you the question: 'How important is it that you get energy again?' P: Very important. (…) Because I've always been energetic. Very important.* (*P2*)

Other participants had difficulties relating the goals to their own situation. For example:

Item *shortness of breath*, step *1 I'm actually never short of breath. But it is quite important.* (*P25*)

There were also participants who did recognize that a certain goal did not apply for them, but they did not understand how to indicate that in the tool.

Some participants were reluctant to use the options 'not at all important' or 'doesn't apply to me', because they deemed those answers socially undesirable.

Since many older persons have multiple health problems, it is possible that a participant experiences a problem with an item, but is admitted for another health problem. Many participants were able to make this distinction. For example:

Item *moving*, step 1: *Yes, that will never be all right again, I can tell. Does no longer apply at all. Already 30 years ago they said: Mr. B., you have to learn to live with that. And they still say that today. Osteoarthritis, there is nothing you can do about it.* (*P18*)

But for others this distinction was more difficult.

Adaptations

To enhance the general understanding, the following adaptations to the tool were made and tested in new participants:

Several adaptations were made in the instruction text.

For peer review only

In the columns with the answer options the word 'important' was added to all answer options. For example: 'very' was changed into 'very important', to make clear that the question was not to evaluate current function, but to indicate how important the goal was.

The sequence of the questions was changed. To enable participants to relate the goals to their own situation, the questions related to somatic complaints were moved to the beginning of the questionnaire.

Another adaptation made to improve the understanding to relate the goals to the patients' own situation, was to add the word 'again' to the goals, to make clear that it is something they had before and they have to regain by the hospital admission. For example: *How important is it to you that you have normal bowel movements again*.

The next adaptation was to move the answer option 'doesn't apply to me' from the last to the first column. This made it easier to find that option.

The final adaptation to improve making the connection between the hospital admission and their goals, was repeating the question in every line. Instead of having the text '*How important is it to you that by this hospital admission…'* on top of the page alone, this question was repeated in every row. Apart from this, several adaptations were made to the layout in order to ease the reading for participants.

Understanding and reactions regarding individual items

The following individual items caused discussion: take pleasure in eating, to know the cause of your complaints, take a short break, and remain alive.

Take pleasure in eating

Some participants had a more epicurean association with this item. Therefore, it was changed into: 'regain appetite'.

Cause of complaints

With the item 'how important is it for you that you know the cause of your complaints?' some participants spontaneously started to describe risk factors like smoking. By changing the item into 'knowing what is wrong with you', this was solved.

Take a short break

The item 'can take a short break' gave many different interpretations, often without any relationship with the hospital. Several alternatives were tried: 'to recharge', 'to take a moment', but these did not improve the understanding. It was therefore decided to remove this item.

For peer review only

Remain alive

The item 'remain alive' gave mixed reactions. Some were irritated by the question. For others it was obvious that it was very important to them that they wanted to remain alive, by adding words like 'of course!'. However, there were also participants who deemed remaining alive less obvious and started to think about the question. Unless the mixed reactions to this question, it was remained because it was not obvious for all participants and because the researchers considered it unreasonable to have a questionnaire with many potential outcomes, but to omit the one outcome that for many participants is considered as the most important.

Additions

Participants gave the following suggestions which were added to the questionnaire: family life, driving, hobbies, urinating. The adaptations and additions led to P-BAS HOP Version 2.

General evaluation

Many participants stated that the questionnaire was quite easy to fill out, although this was not always congruent with the observations about their understanding. Several mentioned enjoying filling in the questionnaire. One participant mentioned that the tool was very important for him in order to state his own priorities. For another participant the questionnaire was considered emotional, because the questions were confronting and he was afraid that many goals were not feasible. For some the questionnaire was somewhat tiring.

Content Validity

The goals the participants mentioned in their own words, were qualified in the questionnaire as at least 'somewhat important' in almost all cases. For example:

> *Yes, that is the quality of life .. Yes, it is important that comes up to standard again. (..) Well, cycling that, that comes in second place. I think* ***walking*** *is more important than ehm .. (…) I have been a volunteer for more than forty years now, helping people fill out tax forms. I think that is important to me. And that is, that is, that is also the* ***volunteer work****. If it is somewhat possible I would like to do that again. (…) Ehm,* ***go on outings****. I would like to keep doing things like that. (P14)*

This participant filled in in the questionnaire: Walking: quite important, (volunteer) work: moderately important, go on outings: moderately important.

TSTI evaluation questionnaire

For peer review only

Sample evaluation questionnaire

Ten patients participated in the TSTI for the evaluation questionnaire at discharge. The sampling of the participants continued until the last version of the questionnaire was considered clear and did not reveal any new problems. Characteristics of the participants are displayed in the third column of Table 1.

Process of testing and adaptations

The process of testing and adapting the evaluation questionnaire was much faster, because many problems with layout and wording of individual items had already been solved in the baseline phase. In the first version, the wording appeared to be too complicated for some participants. Therefore, the original formulation: 'The hospitalisation helped me to….' Was changed into: 'Because of the hospitalisation….'. This adaptation was clear for all the following participants and led to Version 2.

4. Field test with Version 2. Item reduction based on mean impact score and correlation.

Version 2 was tested with a new group of hospitalised older patients. The aim of this field test was to assess the feasibility of the P-BAS HOP in combination with other questionnaires. The trained research assistants observed during the field test that the tool was too time consuming and that some patients still had difficulties relating the questions to their personal situation, as was observed in the TSTI. Therefore, the following extra adaptations were made: item reduction, answer option reduction, and splitting the tool into two phases.

Participants

Eligible participants were consecutive patients aged 70 years and older; planned or unplanned hospitalised on medical or surgical wards of a university teaching hospital, expected to stay for at least 48 hours; and at maximal four days hospitalised at the moment of interviewing; able to speak and understand Dutch and were without cognitive impairment. Inclusion criteria were verified with the staff nurse. Patients were approached by a trained research assistant and gave signed informed consent to participate. The questionnaire was then conducted in a face to face interview with the research assistant, but to patients in a better condition and with middle or higher education the opportunity was given to fill in the questionnaire themselves, an option which only a minority of patients choose.

Item reduction

For peer review only

As this is a formative tool, item reduction procedures suitable for reflective tools, such as based on factor analysis and Cronbach's alpha, are not relevant.[14] Item reduction was therefore based on correlation and mean impact score.

Items within one category with a strong correlation, measured probably the same construct. Therefore, from dyads with a Spearman's rank-order correlation > 0.7, one item was removed.[14] For the calculation of the Spearman's rank-order correlation coefficient, the answer option 'does not apply to me now' and 'not at all important' were coded as 0, the options somewhat, moderately, quite important and very important were coded respectively as 1, 2, 3, 4.

For the reduction based on mean impact score, all items were sorted into categories. For each item the mean impact score was calculated: [% for whom the item played a role] * [mean importance for that item]. From every category with two or more items, the item with the lowest mean impact score was removed.[14] The field test was repeated in a regional teaching hospital by a trained research assistant, to check whether the impact differed in another context.

Results

The Benefit Assessment Scale Version 2 consisted of 32 items. In the three month inclusion period, 492 consecutive eligible patients meeting the inclusion criteria were admitted on the selected wards. Of these patients, 238 were not approached for logistic reasons, for example the patient could not be interviewed within the first four days because of absence for treatment, transfer from ward, shortage of research assistants. Hence, 254 patients were approached for informed consent and 106 patients (42%) gave informed consent. Of the 106 included patients, the P-BAS was not administered 15 times because of lack of time (for example patient had to leave for treatment or discharge) or the patient was too tired. This resulted in 91 administered P-BAS questionnaires. Of the 91 participants, 20 answered the questionnaire independently written and 71 were interviewed by the research assistant. Characteristics of the participants are displayed in Table 2 and the results are shown in Table 3.

Table 2. Participants Field test (n=91)

Characteristic	n
Gender	
Male	63
Female	28
Age (years), median (range)	75 (70 – 96)
Native language	

For peer review only

Dutch	55
Local dialect	27
Frisian	3
Unknown	6
Educational level*	
Low	22
Middle	47
High	22
Specialty	
Medical	42
Surgical	23
Cardiology	26
Admission type	
Acute	60
Elective	31

* Definition educational level: Low= no education, primary school, basic vocational training; Middle = secondary education, vocational training; High = bachelor, master

Table 3. Scores of Version 2 Benefit Assessment Scale Baseline. (n=91)

				Importance				
	Missing		Does not apply to me now n (%)	Not at all n (%)	Some-what n (%)	Moderately n (%)	Quite n (%)	Very n (%)
Item	Failed* n (%)	n.d.† n (%)						
Better	1 (1.1)	0	8 (8.8)	0	1 (1.1)	2 (2.2)	17 (18.7)	62 (68.1)
Weight	1 (1.1)	1 (1.1)	57 (62.6)	10 (11.0)	4 (4.4)	7 (7.7)	3 (3.3)	8 (8.8)
Condition	0	0	17 (18.7)	2 (2.2)	1 (1.1)	6 (6.6)	31 (34.1)	34 (37.4)
Energy	0	1 (1.1)	18 (19.8)	1 (1.1)	0	3 (3.3)	33 (36.3)	35 (38.5)
Pain	1 (1.1)	1 (1.1)	33 (36.3)	0	2 (2.2)	4 (4.4)	9 (9.9)	41 (45.1)
Bowel movements	0	1 (1.1)	58 (63.7)	4 (4.4)	2 (2.2)	1 (1.1)	12 (13.2)	13 (14.3)
Urinate	0	1 (1.1)	64 (70.3)	4 (4.6)	0	1 (1.1)	10 (11.0)	11 (12.1)
Shortness of breath	1 (1.1)	0	39 (42.9)	1 (1.1)	2 (2.3)	5 (5.5)	11 (12.1)	32 (35.2)
Walking	0	1 (1.1)	32 (35.2)	1 (1.1)	3 (3.3)	5 (5.5)	16 (17.6)	33 (36.3)
Moving	0	1 (1.1)	35 (38.5)	1 (1.1)	2 (2.2)	5 (5.5)	18 (19.8)	29 (31.9)
Appetite	0	2 (2.2)	55 (60.4)	1 (1.1)	2 (2.2)	6 (6.6)	9 (9.9)	16 (17.6)
Knowing what is	0	1 (1.1)	32 (35.2)	2 (2.2)	2 (2.2)	1 (1.1)	12 (13.2)	41 (45.1)

For peer review only

wrong								
Disease under control	0	1 (1.1)	10 (11.0)	0	0	2 (2.2)	15 (16.5)	63 (69.2)
Alive	0	1 (1.1)	2 (2.2)	2 (2.2)	1 (1.1)	1 (1.1)	13 (14.3)	71 (78.0)
Enjoy	0	2 (2.2)	20 (22.0)	0	0	2 (2.2)	13 (14.3)	54 (59.3)
Freedom	0	1 (1.1)	31 (34.1)	0	1 (1.1)	1 (1.1)	12 (13.2)	45 (49.5)
Cooking	0	2 (2.3)	51 (56.0)	1 (1.1)	4 (4.4)	2 (2.2)	14 (15.4)	17 (18.7)
Housework	0	1 (1.1)	51 (56.0)	2 (2.2)	5 (5.5)	5 (5.5)	10 (11.0)	17 (18.7)
Groceries	0	1 (1.1)	42 (46.2)	1 (1.1)	5 (5.5)	9 (9.9)	12 (13.2)	21 (23.1)
Wash and dress	0	2 (2.3)	51 (56.0)	0	0	2 (2.2)	14 (15.4)	22 (24.2)
Garden	0	1 (1.1)	48 (52.7)	2 (2.2)	1 (1.1)	8 (8.8)	10 (11.0)	21 (23.1)
Sports	0	1 (1.1)	46 (50.5)	7 (7.7)	2 (2.2)	9 (9.9)	7 (7.7)	19 (20.9)
Hobbies	0	1 (1.1)	39 (42.9)	1 (1.1)	2 (2.2)	4 (4.4)	13 (14.3)	31 (34.1)
Work	0	3 (3.3)	63 (69.2)	3 (3.3)	0	4 (4.4)	7 (7.7)	11 (12.1)
Driving	0	2 (2.2)	46 (50.2)	1 (1.1)	0	2 (2.2)	8 (8.8)	32 (35.2)
Outings	0	1 (1.1)	28 (30.8)	1 (1.1)	3 (3.3)	8 (8.8)	19 (20.9)	31 (34.1)
Visiting	0	3 (3.3)	28 (30.8)	1 (1.1)	4 (4.4)	6 (6.6)	19 (20.9)	30 (33.0)
Family life	0	4 (4.4)	39 (42.9)	0	0	2 (2.2)	12 (13.2)	34 (37.4)
Home	0	2 (2.3)	21 (23.1)	1 (1.1)	0	1 (1.1)	4 (4.4)	62 (68.1)
Independence	1 (1.1)	3 (3.3)	29 (31.9)	0	1 (1.1)	2 (2.2)	6 (6.6)	49 (53.8)

* Measurement failed: invalid answer due to two options filled in

† n.d. = not done. No answer was given.

As seen in Table 3, the number of missing values ranges from zero to four per item. The answer options with the lowest priorities were used the least, especially 'not at all important' and 'somewhat important'. Therefore, and also because on reflection the options 'somewhat' and 'moderately' were very close, we decided to remove the option 'moderately'.

Four dyads had a Spearman's rank-order correlation coefficient > 0.7: energy and condition (r_s = .80); moving and walking (r_s = .87); cooking and groceries (r_s = .75); cooking and housekeeping (r_s = .70) Therefore of these dyads, one item was removed (condition, moving and cooking), inspired by the information derived from the TSTI's.

Table 4 shows the items with mean impact scores, sorted per category and descending mean impact scores. From the categories with at least two items, the item with the lowest mean impact score was removed. To give participants still the opportunity to indicate their individual priorities, even when being a minority, we added an open option to add extra individual goals.

Table 4. Mean impact scores per category.

	University hospital			Regional teaching hospital		
**Goals**	**Applied**	**Importance**	**Mean**	**Applied**	**Importance**	**Mean**

For peer review only

	(%)	score (M)	impact score	(%)	score (M)	impact score
**Remain alive**						
Remain alive	98	3.70	3.62	75	2.64	1.90
**Controlling disease**						
Controlling disease	89	3.76	3.34	29	2.43	0.70
**Improving condition**						
Feeling better	91	3.71	3.38	71	2.73	1.94
Energy	80	3.40	2.72	50	2.23	1.12
Condition	*81*	*3.27*	*2.66*	*65*	*2.34*	*1.53*
Weight	*36*	*1.84*	*0.66*	*9*	*2.33*	*0.20*
**Alleviating complaints**						
Pain	63	3.59	2.26	44	2.72	1.20
Breath	57	3.39	1.92	38	2.64	0.99
Appetite	38	3.09	1.18	35	2.39	0.83
Bowel	35	2.88	1.03	29	2.47	0.71
Urinate	*29*	*2.92*	*0.83*	*17*	*2.67*	*0.46*
**Enjoying life**						
Enjoying life	78	3.75	2.91	31	2.53	0.78
**Improving/maintaining social functioning**						
Outing	69	3.23	2.23	27	2.11	0.57
Visiting	68	3.22	2.20	21	1.91	0.40
Family life	*55*	*3.67*	*2.03*	*5*	*2.80*	*0.13*
**Knowing what is wrong**						
Wrong	64	3.52	2.27	39	2.58	0.99
**Regaining/maintaining independence, freedom**						
Home	76	3.85	2.94	15	2.50	0.39
Independence	67	3.78	2.52	17	2.44	0.42
Freedom	*66*	*3.71*	*2.43*	*23*	*2.54*	*0.59*
**Improving daily functioning**						
Walking	64	3.33	2.14	54	2.57	1.38
Moving	*61*	*3.31*	*2.02*	*34*	*2.54*	*0.86*
Driving	48	3.63	1.75	14	2.13	0.31
Groceries	53	2.98	1.59	19	2.30	0.44
Wash/dress	43	3.53	1.52	26	2.52	0.65
Cooking	*43*	*3.11*	*1.33*	*15*	*1.75*	*0.27*
Housework	*43*	*2.90*	*1.26*	*20*	*1.86*	*0.38*
**Resuming work/hobbies**						
Hobbies	57	3.39	1.92	20	2.00	0.40
Garden	47	3.12	1.46	16	1.24	0.20
Sports	49	2.66	1.30	23	1.58	0.37
Work	*28*	*2.92*	*0.83*	*13*	*1.92*	*0.24*

The removed items are indicated in italic.

Repetition field test in regional teaching hospital

The field test was repeated in a regional teaching hospital with the same items, but with fewer answer options and the questions in two steps, as explained in the next paragraph. In the eight week inclusion period, 209 patients meeting the inclusion criteria were admitted on the wards. Of these patients, 56 were not approached for logistic reasons. 153 Were therefore approached for informed consent and 104 patients (67%) gave informed consent. The items with the lowest mean impact scores were the same for most categories, except for the categories independence/freedom, improving daily functioning and work/hobbies.

Splitting tool into two phases

Since some problems with understanding remained, especially the difficulties relating the goals to their own situation, as described in the TSTI, we decided to split the tool into two phases. In the first phase an inventory of subjects with problems or limitations was made. These could be problems/limitations at the moment of interview, at admission, or expected problems/limitations. In the second phase, only the importance was asked for the goals related to the subjects that applied. As this adaptation complicated the tool, we decided to use it as an interview-based tool. The item reduction and splitting into two phases, resulted in P-BAS HOP version 3.

5. TSTI with Version 3.

Version 3 was tested again with the TSTI in hospitalised older patients. The procedure was identical as in step 3, however as this version is only applicable as an interview version, this was done with an interviewer and observant. The observant only observed during the first step, and took over the interview role in the second and third steps.

Results

Eight participants participated in the TSTI about Version 3. Characteristics of the participants are displayed in the last column of Table 1.

General understanding

In general, the tool in two phases was well-understood. For example:

> Item *shortness of breath*, phase 1, step 1: *No, I have no problems with that, you know, shortness of breath.* (*A1*)
>
> Or:

For peer review only

> Item *shortness of breath*, phase 1, step 1: *Yes, that is present! And for that reason, I am admitted here. My oxygen was too low. And my carbon dioxide level is not good, much too high. Yes, complication of, yes.* (*A3*)

We shortened the instructions, but did not modify the content of the tool. This last adaptation led to the final questionnaire (Appendix 2). The completion of this baseline questionnaire took 5 to 24 minutes, with a median of 11 minutes.

DISCUSSION

The Patient Benefit Assessment Scale for Hospitalised Older patients (P-BAS HOP) was constructed as a tool that should be capable both to identify the goals and priorities of the individual older hospitalised patient and to measure the outcomes relevant to him regarding hospitalisation.

The items of the P-BAS HOP were based on interviews with hospitalised older patients. Including patients in the generation of patient reported outcomes is not self-evident and is even absent in many cases.[18] But even when patients are involved in the generation of outcomes, they still only reflect the priorities of the overall patient population and not the individual patient. Therefore, the major advantage of the P-BAS HOP is that patients can indicate their individual priorities, which also leads to individual benefit-scores.

Indicating individual priorities is also possible with the GAS, but the GAS is more time consuming, varying from 15-20 minutes for experienced assessors,[19] to 90 minutes per patient,[20] while the P-BAS HOP takes 5 to 24 minutes, with a median of 11 minutes. Moreover, for some older patients it might be difficult to formulate their own goals,[12] and the P-BAS HOP helps patients with examples of predefined goals.

More recently, models for goal based decision making were developed,[21-23] but these methods are more suitable for clinical encounters to align treatment option with patient goals. The major advantage of the P-BAS HOP is that it is a more suitable and efficient tool to measure personalised outcomes in, for example, trials. It also could replace a diversity of existing tools, since it covers several dimension like symptoms, daily functioning, social functioning. Examples for which the P-BAS HOP could be used are to compare the personalised outcomes for alternatives of hospital admission, such as,[24-27] the effectiveness of better geriatric management of in-hospital patients,[28] or in a narrower way, to compare the effectiveness of different treatment methods on personalised outcomes.

The pilot and field tests of the P-BAS HOP started already before we achieved complete saturation of goals in the qualitative interviews. Therefore, patients had the possibility to add goals during the TSTI. Several goals were added during the TSTI, which also appeared later in the qualitative

For peer review only

interviews.[3] Still, the qualitative interviews revealed later some extra target complaints, which were not included in the P-BAS HOP, such as vomiting, dizziness and sweating. Yet, in the final version of the P-BAS HOP, patients still have the opportunity to add personal goals which were not mentioned before.

By using the mean impact score to reduce items, items considered least important by the overall sample were removed, though this does not take account of the priorities of individuals who deviate from the majority. For this reason the extra open option was added. Most removed items, based on mean impact score, were confirmed when repeated in the regional teaching hospital. The only exceptions were in the categories *improving daily functioning*, *resuming work/hobbies,* and *regaining/maintaining independence/freedom.*

In the categories improving daily functioning and resuming work/hobbies, the lowest priorities were 'housework' and 'work' in the first sample and 'driving' and 'gardening' in the second. Since driving and work were the second lowest priority in the second sample, the removal of housework and work could be justified.

In the category *regaining/maintaining independence/freedom* priorities in both hospitals were entirely opposite. We therefore have to conclude that we were too early to remove the item freedom. It is unclear whether these differences are caused by different contexts or because the field test in the regional hospital was after splitting the questionnaire into two phases and therefore the questions were altered.

Limitations

The P-BAS HOP is only tested in hospitalised patients without cognitive impairment. It is therefore unknown if it is suitable in other contexts and it might be too complex for patients with cognitive impairment. In addition, the P-BAS HOP is only tested in the Netherlands and the translated English version has not yet been tested. Therefore, it is unknown whether the P-BAS HOP is applicable in other languages and cultures.

The TSTI gave valuable insights into the understanding of the questionnaire and the completion behaviour of the participants. Many adaptations were made, but it proved difficult to make the questionnaire understandable for all patients. These kinds of difficulties were seen in various examples where the TSTI was used.[16, 29-31] Unfortunately, the final version is only suitable to be completed with an interviewer and not as a self-administered questionnaire. The TSTI gave a first indication of the content validity, but further quantitative research into the construct validity, in which the priority of goals can be compared with experienced symptoms or limitations at admission and the achievement of goals can be compared with progression or deterioration of other constructs, test-retest reliability of baseline and evaluation questionnaire and responsivity to test the validity of the PBI is needed.

For peer review only

Conclusions

The Patient Benefit Assessment Scale for Hospitalised Older Patients (P-BAS HOP) is a potentially suitable interview-based tool to identify the priorities and relevant outcomes of the individual older hospitalised adult. Based on these data it is possible to compute an individual Patient Benefit Index, which is an overall value between 0 (no benefit) and 3 (maximal benefit), which reflects the achievement of the goals weighted by the importance. Further quantitative research is needed to investigate the construct validity, reliability and responsiveness.

Acknowledgements We would like to thank all patients who participated in the interviews for sharing their stories with us and their feedback on the different versions of the P-BAS HOP. We thank the panel members for their feedback on the draft version. We would also like to thank all the research assistants and students for the many interviews they conducted, especially Gerdine Boot for all the patients she interviewed in the regional teaching hospital. We would like to thank Daniël Bosold, Janet Vroomen, Caroline Mutsaers, Margaretha Schenkeveld and Anna Pot for their role in the translation process of the P-BAS HOP.

Funding This study was funded by an unrestricted grant from the Dutch Foundation for Effective Elderly Care and the University of Groningen.

Author's contribution MJvdK designed the study. MJvdK conducted the Three Step Test Interviews (TSTI's). MJvdK coded the data of the TSTI's. MJvdK and SEdR regularly discussed the codes and the progress and adaptations of the different versions of the P-BAS HOP. SEdR supervised the field test in the University hospital, BCvM in the Regional teaching hospital. MJvdK wrote the first draft of the manuscript, GJD, BCvM and SEdR contributed significantly to subsequent manuscript revisions. All authors have read and approved the final version of the manuscript.

Competing interests None declared

Data sharing All data relevant to the study are included in the article

Patient consent All patients gave informed consent

Ethical approval

The Medical Ethics Research Committee of the UMCG (file number M16.192615 and M16.199647) confirmed that the Medical Research Involving Human Subjects Act did not apply to the research project. Official approval by the committee was therefore not required.

REFERENCES

For peer review only

1 Reuben DB, Tinetti ME. Goal-oriented patient care--an alternative health outcomes paradigm. *N Engl J Med* 2012;366:777-9 doi:10.1056/NEJMp1113631.

2 Tinetti ME, Naik AD, Dodson JA. Moving from disease-centered to patient goals-directed care for patients with multiple chronic conditions: Patient value-based care. *JAMA Cardiology* 2016;1:9-10 doi:10.1001/jamacardio.2015.0248.

3 Van der Kluit MJ, Dijkstra GJ, de Rooij S,E. Goals of older hospitalised patients: a qualitative descriptive study. *BMJ open* 2019;9:e029993- doi:10.1136/bmjopen-2019-029993.

4 Vermunt NP, Harmsen M, Elwyn G, et al. A three-goal model for patients with multimorbidity: A qualitative approach. *Health Expect* 2018;21:528-38 doi:10.1111/hex.12647 [doi].

5 Donnelly C, Carswell A. Individualized outcome measures: a review of the literature. *Can J Occup Ther* 2002;69:84-94.

6 Mangin D, Stephen G, Bismah V, et al. Making patient values visible in healthcare: a systematic review of tools to assess patient treatment priorities and preferences in the context of multimorbidity. *BMJ Open* 2016;6:e010903- doi:10.1136/bmjopen-2015-010903.

7 van Seben R, Reichardt L, Smorenburg S, et al. Goal-Setting Instruments in Geriatric Rehabilitation: A Systematic Review. *J Frailty Aging* 2017;6:37-45 doi:10.14283/jfa.2016.103.

8 Fried TR, Tinetti M, Agostini J, et al. Health outcome prioritization to elicit preferences of older persons with multiple health conditions. *Patient Educ Couns* 2011;83:278-82 doi:10.1016/j.pec.2010.04.032.

9 Stolee P, Awad M, Byrne K, et al. A multi-site study of the feasibility and clinical utility of Goal Attainment Scaling in geriatric day hospitals. *Disabil Rehabil* 2012;34:1716-26 doi:10.3109/09638288.2012.660600 [doi].

10 Battle CC, Imber SD, Hoehn-Saric R, et al. Target complaints as criteria of improvement. *Am J Psychother* 1966;20:184-92 doi:10.1176/appi.psychotherapy.1966.20.1.184 [doi].

11 Hesbacher PT, Rickels K, Weise C. Target symptoms: a promising improvement criterion in psychiatric drug research. *Arch Gen Psychiatry* 1968;18:595-600.

12 Schulman-Green DJ, Naik AD, Bradley EH, et al. Goal setting as a shared decision making strategy among clinicians and their older patients. *Patient Educ Couns* 2006;63:145-51 doi:S0738-3991(05)00275-2 [pii].

13 Augustin M, Reich C, Schaefer I, et al. Development and validation of a new instrument for the assessment of patient-defined benefit in the treatment of acne. *J Dtsch Dermatol Ges* 2008;6:113-20 doi:10.1111/j.1610-0387.2007.06540.x.

14 De Vet HCW, Terwee CB, Mokkink LB, et al. Measurement in Medicine. A Practical Guide. Cambridge: Cambridge University Press 2011.

15 Beaton DE, Bombardier C, Guillemin F, et al. Guidelines for the Process of Cross-Cultural Adaptation of Self-Report Measures. *Spine* 2000;25:3186-91 doi:10.1097/00007632-200012150-00014.

For peer review only

16 Pool JJM, Hiralal S, Ostelo, Raymond W J G, et al. Added value of qualitative studies in the development of health related patient reported outcomes such as the Pain Coping and Cognition List in patients with sub-acute neck pain. *Man Ther* 2010;15:43-7 doi:10.1016/j.math.2009.05.010.

17 Van Der Veer K, Ommundsen R, Hak T, et al. Meaning Shift of Items in Different Language Versions. A Cross-National Validation Study of the Illegal Aliens Scale. *Quality and Quantity* 2003;37:193-206.

18 Wiering B, de Boer D, Delnoij D. Patient involvement in the development of patient-reported outcome measures: a scoping review. *Health Expect* 2017;20:11-23 doi:10.1111/hex.12442 [doi].

19 Stolee P, Rockwood K, Fox RA, et al. The use of goal attainment scaling in a geriatric care setting. *J Am Geriatr Soc* 1992;40:574-8.

20 Toto PE, Skidmore ER, Terhorst L, et al. Goal Attainment Scaling (GAS) in geriatric primary care: a feasibility study. *Arch Gerontol Geriatr* 2015;60:16-21 doi:10.1016/j.archger.2014.10.022 [doi].

21 Elwyn G, Vermunt NPCA. Goal-Based Shared Decision-Making: Developing an Integrated Model. *Journal of Patient Experience* 2019:1-9 doi:10.1177/2374373519878604.

22 Naik AD, Dindo LN, Van Liew JR, et al. Development of a Clinically Feasible Process for Identifying Individual Health Priorities. *J Am Geriatr Soc* 2018;66:1872-9 doi:10.1111/jgs.15437 [doi].

23 Tinetti ME, Naik AD, Dindo L, et al. Association of Patient Priorities-Aligned Decision-Making With Patient Outcomes and Ambulatory Health Care Burden Among Older Adults With Multiple Chronic Conditions: A Nonrandomized Clinical Trial. *JAMA Intern Med* 2019;179:1688-97 doi:10.1001/jamainternmed.2019.4235 [doi].

24 Pouw MA, Calf AH, van Munster BC, et al. Hospital at Home care for older patients with cognitive impairment: a protocol for a randomised controlled feasibility trial. *BMJ Open* 2018;8:e020332,2017-020332 doi:10.1136/bmjopen-2017-020332 [doi].

25 Ribbink ME, Macneil-Vroomen JL, van Seben R, et al. Investigating the effectiveness of care delivery at an acute geriatric community hospital for older adults in the Netherlands: a protocol for a prospective controlled observational study. *BMJ Open* 2020;10:e033802,2019-033802 doi:10.1136/bmjopen-2019-033802 [doi].

26 Shepperd S, Iliffe S, Doll H, et al. Admission avoidance hospital at home. *Cochrane Database Syst Rev* 2016;9:CD007491- doi:10.1002/14651858.CD007491.pub2.

27 Shepperd S, Cradduck-Bamford A, Butler C, et al. A multi-centre randomised trial to compare the effectiveness of geriatrician-led admission avoidance hospital at home versus inpatient admission. *Trials* 2017;18:491,017-2214-y doi:10.1186/s13063-017-2214-y [doi].

28 Van Grootven B, Flamaing J, Dierckx de Casterlé B, et al. Effectiveness of in-hospital geriatric co-management: a systematic review and meta-analysis. *Age Ageing* 2017;46:903-10 doi:10.1093/ageing/afx051 [doi].

29 Jansen H, Hak T. The Productivity of the Three-step Test-interview (TSTI) Compared to an Expert Review of a Self-administered Questionnaire on Alcohol Consumption. *Journal of official statistics* 2005;21:103-20.

For peer review only

30 Liu RD, Buffart LM, Kersten MJ, et al. Psychometric properties of two physical activity questionnaires, the AQuAA and the PASE, in cancer patients. *BMC Med Res Methodol* 2011;11:30,2288-11-30 doi:10.1186/1471-2288-11-30 [doi].

31 van Alphen A, Westerman MJ, Visschedijk JH, et al. A qualitative study of Falls Efficacy Scale-International/Hips. What do we measure?. *Tijdschr Gerontol Geriatr* 2013;44:3-11 doi:10.1007/s12439-013-0005-9 [doi].

For peer review only

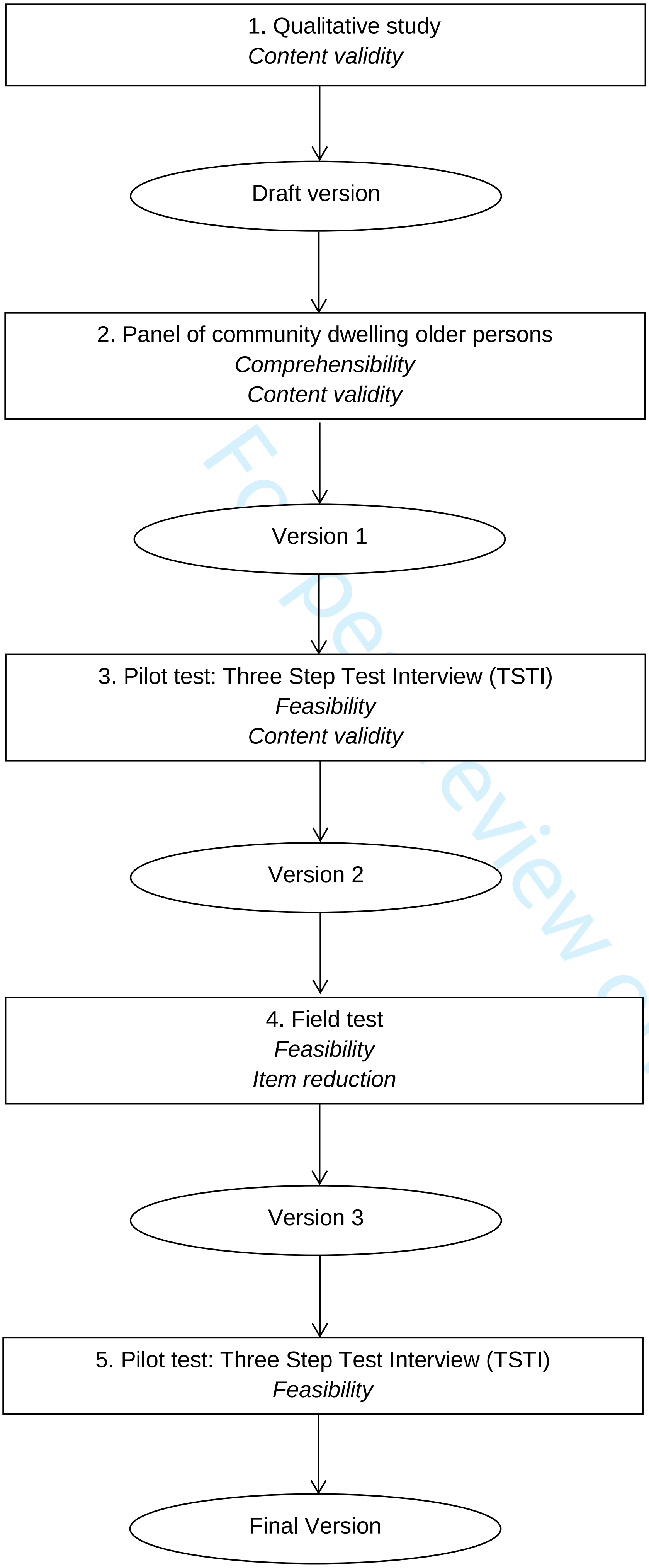

For peer review only - http://bmjopen.bmj.com/site/about/guidelines.xhtml

Appendix 1. Version 1. Patient Benefit Assessment Scale

Hospitalisation goals

The following questions cover how important the goals below are for you during your current hospitalisation.

Can you indicate how important each goal below is for you? You can choose from: 'not at all', 'somewhat', 'moderately' 'quite', or 'very'. If a goal doesn't apply to you, for example because you don't have difficulty with the listed problem (for example with bowel movements, or shortness of breath) or because you don't have a garden, then choose 'does not apply to me'.

	How important is it for you that through this hospitalisation…	Not at all	Somewhat	Moderately	Quite	Very	Does not apply to me
1	You feel better						
2	You regain weight						
3	Your condition improves						
4	You have more energy						
5	You can walk better						
6	You move easier						
7	You can do housework						
8	You can cook						
9	You can do the groceries						
10	You can garden						
11	You can take pleasure in eating						
12	You can wash and dress yourself						
13	You can exercise or participate in sports						
14	You have no pain						
15	You have normal bowel movements						
16	You have less shortness of breath						
17	Your disease is under control						
18	You remain alive						
19	You can enjoy life						
20	You regain your freedom						
21	You can resume your (volunteer) work						
22	You can go on outings						
23	You can visit friends or family						
24	You can take a short break						
25	You know the cause of your complaints						
26	You can go back home						
27	You regain your independence						

For peer review only

Evaluation of hospitalisation goals

In the beginning of your hospitalisation you indicated how important various goals for you were. Can you indicate for each of the goals below how much the hospitalisation has helped to achieving the goal? You can indicate whether the hospitalisation helped you 'not at all', 'somewhat', 'moderately' 'quite', or 'very'. If a goal didn't apply to you, for example because you didn't have difficulty with the listed problem (for example with bowel movements, or shortness of breath) or because you have no garden, then choose 'did not apply to me'.

	The hospitalisation helped me to...	Not at all	Somewhat	Moderately	Quite	Very	Did not apply to me
1	Feel better						
2	Regain weight						
3	Improve my condition						
4	Have more energy						
5	Walk better						
6	Move easier						
7	Do the housework						
8	Cook						
9	Do the groceries						
10	Garden						
11	Take pleasure in eating						
12	Wash and dress myself						
13	Exercise or Participate in sports						
14	Have no pain						
15	Have normal bowel movements						
16	Have less shortness of breath						
17	Keep my disease under control						
18	Remain alive						
19	Enjoy life						
20	Regain my freedom						
21	Resume my volunteer work						
22	Go on outings						
23	Visit family or friends						
24	Can take a short break						
25	Know what the cause of my complaints is/was						
26	Go back to my home						
27	Regain my independence						

For peer review only

Appendix 2. Final Version Patient Benefit Assessment Scale

Hospitalisation Goals

People differ in what they wish to achieve with a hospitalisation. They have different goals. This depends on what they suffer from, what they are hospitalised for, and what they find important in life.

I am now going to mention some subjects that may be important to you during this hospitalisation. Can you say whether each applies to you?

A subject applies to you if you experience or anticipate problems or limitations and this applies to your life. For people who, for example, are short of breath, the subject shortness of breath probably applies, but not for others. For others they may be struggling to enjoy life because of their illness, but if you experience no problems with it, then this doesn't apply to you.

Instruction for the interviewer: Circle the number for the subject that applies to the participant.

If a participant asks what you mean by a subject, say:

Are you experiencing problems with [subject] now, or when you were admitted, or are you expecting problems with it because of your illness or hospitalisation?

For the functional subjects, prefer not to use the word 'problem', but rather 'trouble' or 'limitation'.

Now follow the subjects, you should indicate whether each subject applies to you or not:

1. Wanting to feel better

Explanation if necessary: do you feel sick or miserable and would you like to feel better?

2. Energy
3. Pain
4. Bowel movements
5. Shortness of breath
6. Walking
7. Appetite
8. Unclarity about what is wrong with me

Explanation if necessary: you may be wondering what is wrong with you. If this was already clear for you when you were admitted then this does not apply.

9. Controlling my disease

Explanation if necessary: Do you suffer from a disease that needs to be controlled?

10. Remain alive

Explanation if necessary: Was your life in danger when you were admitted or do you think that this hospitalisation must contribute to remain alive?

11. Enjoying life
12. Housework
13. Groceries
14. Washing and dressing myself
15. Gardening
16. Exercise or Sports
17. Hobbies
18. Driving
19. Going on outings
20. Visiting family or friends

For peer review only

21. Return back to my home

Explanation if necessary: Are you unsure whether you can return to your own home?

22. Independence

23. Are there other themes which are important to you during this hospitalisation that I have not mentioned yet? If so, which?

Now that we have identified the themes that apply to you, I am going to ask to what extent they are goals during this hospitalisation.

Can you indicate for each goal that I mention how important it is during this hospitalisation? You can choose from 'not at all important', 'moderately important' 'quite important', or 'very important'.

For some goals you may still say that they do not apply, for example because they do not matter during this hospitalisation. Then you indicate "does not apply to me now".

Instruction to interviewer: Read only the goals which you have circled.

For peer review only

		does **not apply** to me now	**not at all** important	**somewhat** important	**quite** important	**very** important
1	How important is it for you that you **feel better again** as a result of this hospitalisation	☐	☐	☐	☐	☐
2	How important is it for you that you **have more energy** as a result of this hospitalisation	☐	☐	☐	☐	☐
3	How important is it for you that you **have less pain** as a result of this hospitalisation	☐	☐	☐	☐	☐
4	How important is it for you that you **have normal bowel movements again** as a result of this hospitalisation	☐	☐	☐	☐	☐
5	How important is it for you that you **are less short of breath** as a result of this hospitalisation	☐	☐	☐	☐	☐
6	How important is it for you that you can **walk better again** as a result of this hospitalisation	☐	☐	☐	☐	☐
7	How important is it for you that you **regain your appetite** as a result of this hospitalisation	☐	☐	☐	☐	☐
8	How important is it for you that you **know what is wrong with you** as a result of this hospitalisation	☐	☐	☐	☐	☐
9	How important is it for you that **your disease is under control** as a result of this hospitalisation	☐	☐	☐	☐	☐
10	How important is it for you that you **remain alive** as a result of this hospitalisation	☐	☐	☐	☐	☐
11	How important is it for you that you can **enjoy life again** as a result of this hospitalisation	☐	☐	☐	☐	☐
12	How important is it for you that you can **do housework again** as a result of this hospitalisation	☐	☐	☐	☐	☐

For peer review only

		does **not apply** to me now	**not at all** important	**somewhat** important	**quite** important	**very** important
13	How important is it for you that you can **do the groceries again** as a result of this hospitalisation	☐	☐	☐	☐	☐
14	How important is it for you that you **can wash and dress yourself again** as a result of this hospitalisation	☐	☐	☐	☐	☐
15	How important is it for you that you **can garden again** as a result of this hospitalisation	☐	☐	☐	☐	☐
16	How important is it for you that you **can exercise or participate in sports again** as a result of this hospitalisation	☐	☐	☐	☐	☐
17	How important is it for you that you **can exercise your hobbies again** as a result of this hospitalisation	☐	☐	☐	☐	☐
18	How important is it for you that you **can drive again** as a result of this hospitalisation	☐	☐	☐	☐	☐
19	How important is it for you that you **go on outings again** as a result of this hospitalisation	☐	☐	☐	☐	☐
20	How important is it for you that you **can visit family or friends again** as a result of this hospitalisation	☐	☐	☐	☐	☐
21	How important is it for you that you **can return to your own home again** as a result of this hospitalisation	☐	☐	☐	☐	☐
22	How important is it for you that you **regain your independence** as a result of this hospitalisation	☐	☐	☐	☐	☐
23	How important is it for you that you ____________________ as a result of this hospitalisation	☐	☐	☐	☐	☐
24	Do you have any goals I haven't mentioned yet? If so: How important is it to you that_____________________________ as a result of this hospitalisation	☐	☐	☐	☐	☐

Evaluation of hospitalisation goals

In the beginning of your hospitalisation you indicated how important various goals for you were. A goal is something you want to achieve with a hospitalisation. Some goals you may have achieved, others maybe not or not entirely.

Can you indicate for each of the goals below how much the hospitalisation has helped to achieving the goal? You can indicate whether the hospitalisation has helped you 'not at all', 'moderately', 'quite', or 'completely'.

Only the goals which applied at baseline are evaluated with the participant.

		Not at all	Somewhat	Quite	Completely
1	Because of the hospitalisation I **feel better again**	☐	☐	☐	☐
2	Because of the hospitalisation I have **more energy**	☐	☐	☐	☐
3	Because of the hospitalisation I have **no more pain**	☐	☐	☐	☐
4	Because of the hospitalisation I have **normal bowel movements again**	☐	☐	☐	☐
5	Because of the hospitalisation I am **less short of breath**	☐	☐	☐	☐
6	Because of the hospitalisation I **walk better again**	☐	☐	☐	☐
7	Because of the hospitalisation I **regained appetite**	☐	☐	☐	☐
8	Because of the hospitalisation I **know what is/ was wrong with me**	☐	☐	☐	☐
9	Because of the hospitalisation **my disease is under control**	☐	☐	☐	☐
10	Because of the hospitalisation I **remained alive**	☐	☐	☐	☐
11	Because of the hospitalisation I **enjoy life again**	☐	☐	☐	☐
12	Because of the hospitalisation I **do my housework again**	☐	☐	☐	☐
13	Because of the hospitalisation I **do the groceries again**	☐	☐	☐	☐
14	Because of the hospitalisation I **wash and dress myself again**	☐	☐	☐	☐
15	Because of the hospitalisation I **garden again**	☐	☐	☐	☐
16	Because of the hospitalisation I **participate in sports again**	☐	☐	☐	☐

For peer review only

		Not at all	Somewhat	Quite	Completely
17	Because of the hospitalisation I **can resumed my hobbies**	☐	☐	☐	☐
18	Because of the hospitalisation I **drive again**	☐	☐	☐	☐
19	Because of the hospitalisation I **go on outings again**	☐	☐	☐	☐
20	Because of the hospitalisation I **visit family or friends again**	☐	☐	☐	☐
21	Because of the hospitalisation I **am back in my own home**	☐	☐	☐	☐
22	Because of the hospitalisation I **regained independence**	☐	☐	☐	☐
23	Because of the hospitalisation ___________________________	☐	☐	☐	☐

For peer review only
